# A low-cost genomics workflow enables isolate screening and strain-level analyses within microbiomes

**DOI:** 10.1186/s13059-022-02777-w

**Published:** 2022-10-12

**Authors:** Jon G. Sanders, Weiwei Yan, Deus Mjungu, Elizabeth V. Lonsdorf, John A. Hart, Crickette M. Sanz, David B. Morgan, Martine Peeters, Beatrice H. Hahn, Andrew H. Moeller

**Affiliations:** 1 Department of Ecology and Evolutionary Biology, Cornell University, Ithaca, NY USA; 2 Gombe Stream Research Center, Kigoma, Tanzania; 3 Department of Psychology and Biological Foundations of Behavior Program, Franklin and Marshall College, Lancaster, PA USA; 4 Department of Anthropology, Emory University, Atlanta, GA 30322 USA; 5 Lukuru Wildlife Research Foundation, Tshuapa-Lomami-Lualaba Project, BP 2012 Kinshasa, Democratic Republic of the Congo; 6 Department of Anthropology, Washington University in St. Louis, 1 Brookings Drive, Saint Louis, MO USA; 7 Wildlife Conservation Society, Congo Program, Brazzaville, B.P. 14537 Republic of Congo; 8 Lester E. Fisher Center for the Study and Conservation of Apes, Lincoln Park Zoo, Chicago, IL USA; 9 Recherche Translationnelle Appliquée Au VIH Et Aux Maladies Infectieuses, Institut de Recherche Pour Le Développement, University of Montpellier, INSERM, 34090 Montpellier, France; 10 Departments of Medicine and Microbiology, Perelman School of Medicine, University of Pennsylvania, Philadelphia, PA USA

## Abstract

Earth’s environments harbor complex consortia of microbes that affect processes ranging from host health to biogeochemical cycles. Understanding their evolution and function is limited by an inability to isolate genomes in a high-throughput manner. Here, we present a workflow for bacterial whole-genome sequencing using open-source labware and the OpenTrons robotics platform, reducing costs to approximately $10 per genome. We assess genomic diversity within 45 gut bacterial species from wild-living chimpanzees and bonobos. We quantify intraspecific genomic diversity and reveal divergence of homologous plasmids between hosts. This enables population genetic analyses of bacterial strains not currently possible with metagenomic data alone.

**Supplementary Information:**

The online version contains supplementary material available at 10.1186/s13059-022-02777-w.

## Introduction

Microbiota are complex mixtures of organisms, with dozens to hundreds of microbial species sharing genes both through ancestry with closely related strains, as well as through horizontal transfer to distantly related lineages [1–3]. Understanding how genetic variation arises and changes within these communities is critical if we hope to develop useful models of their evolution [4].

But despite the tremendous advances in sequencing technology in the past decades, the paired phenomena of within-species strain diversity and between-species horizontal gene transfer still present a challenge to assessing the genetic structure of populations within diverse metagenomes like the mammalian gut. Community metagenome sequencing can rapidly generate massive quantities of data from a microbiome, but with only limited ability to link genetic changes within the same genome or in populations of closely related cells [5, 6].

Mobile DNA elements, especially plasmids, are even more difficult to place in a metagenomic context [7–9]. In principle, cultivation offers a much more robust way to explore genomic variation within populations. Although cultivation necessarily introduces bias in the specific taxa that are recovered, and thus cannot replace metagenomic methods for understanding microbial communities, it offers a few clear advantages. By confidently drawing cellular bounds around genes, isolation represents a gold standard for describing genomic diversity and a necessary prerequisite for empirically demonstrating the functional consequences of such variation. For this reason, cultivation has seen renewed interest, with automation and screening techniques being employed to increase the breadth of diversity that can be reasonably assessed. However, such high-throughput approaches typically require enormous investments in capital equipment and labor [10–13], putting them out of reach for many researchers. This is especially true for those studying non-model systems where the bulk of unstudied microbial diversity is likely to be found. Advances in miniaturization and microfluidic technologies may one day permit rapid high-throughput cultivation from diverse environments [14, 15], though such approaches are not yet widely available. And while conventional isolation techniques using traditional solid media can easily generate thousands of isolates in a short period of time, generating genomic data from this many isolates is still a major barrier to most laboratories.

The recent availability of distributed, open-source laboratory automation and distributed manufacturing technologies suggests a potential solution: adapting high-throughput genome sequencing techniques to relatively inexpensive commercial and in-house-manufactured equipment. In combination with the extremely low per-base cost of modern sequencing, such an approach offers the potential to realize much of the benefits of capital-intensive conventional high-throughput culturing and sequencing pipelines at a fraction of the required investment.

Motivated by our desire to explore genomic evolution in the microbial populations associated with natural mammalian gut microbiomes, we set out to design an inexpensive end-to-end high-throughput genome sequencing protocol that could be easily replicated with a minimum of capital expenditure. While other high-throughput genomic protocols have been published that can reach low marginal costs per genome [16, 17], they typically rely on expensive high-precision robotics and other specialized equipment, thus making them more suited to well-funded laboratories or core facilities. We developed protocols, 3D-printed custom labware, and analysis pipelines to enable cost-effective high-throughput whole-genome sequencing of natural gut microbiota. These methods allowed us to circumvent traditional 16S rRNA-gene or mass spectrometry-based screening approaches, instead using full-genome sequencing to identify all cultivated isolates. Moreover, this approach enabled the generation and assembly of thousands of bacterial genomes from the hominid gut microbiota rapidly and at low cost relative to existing approaches. Results revealed substantial variation in the distribution of strain-level diversity among wild-living chimpanzees and bonobos (*Pan*), and, importantly, allowed us to link putative plasmids to their specific bacterial hosts across *Pan* individuals, populations, and subspecies.

## Results

For the purposes of validating the workflow, we carried every sample from DNA extraction through to sequencing. Even if, for example, an isolate failed to grow during liquid culture, we did not exclude it from downstream steps. This enabled us to determine appropriate exclusion criteria for future use.

**Fig. 1:**
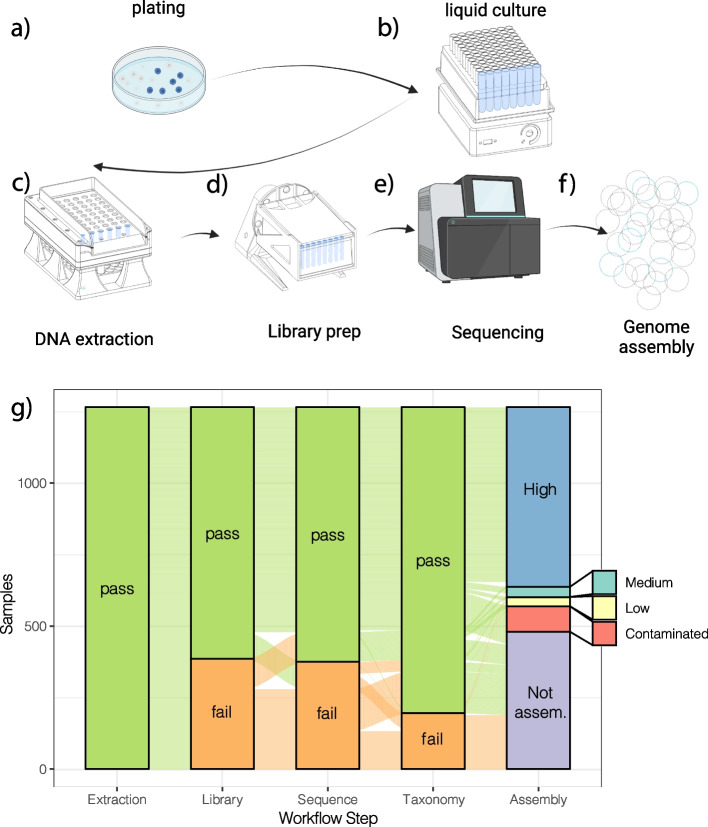
Illustration of isolate genome screening workflow, highlighting 3D-printed labware. **a** Dilution plating on standard media. **b** Liquid culture in 1.8 mL strip tubes, using 3D-printed compact plate shaker to enhance nutrient and gas mixing. **c** DNA extraction on Opentrons OT-2 platform, using 3D-printed bead dispenser to aliquot lysis beads directly into liquid culture tubes. **d** Library prep on Opentrons OT-2 platform, using 3D-printed plate rotator to enhance efficiency of DNA binding to magnetic beads. **e** DNA sequencing on Illumina platform. **f** Genome analysis and assembly. **g** Results from initial rounds of screening, showing samples passing certain QC thresholds at each stage (extraction: 0.1 ng/µL DNA concentration; library prep: 0.5 ng/µL DNA concentration; sequencing: 25 Mbp sequence yield; taxonomy: taxonomy assigned by Sourmash; assembly: high-, medium-, and low-quality assemblies), contaminated assemblies, and unassembled samples. Colored lines connect the same sample through each stage of the chart. Note that even many samples with low DNA extraction concentrations often yielded sufficient sequence data for taxonomic assignment

In total, we picked, grew in liquid culture, extracted DNA from, and sequenced 1879 bacterial isolates (mean of 209, standard deviation of 143 per host individual). Of these, 1265 yielded extractions with DNA concentrations above 0.1 ng/µL; 1049 yielded library concentrations ≥ 0.5 ng/µL; 933 yielded ≥ 25 Mbp of sequence; and 715 yielded high-quality assemblies (> 90% complete and < 5% contaminated), 51 medium-quality assemblies (> 50% complete and < 5% contaminated), and 50 low-quality assemblies (≤ 50% complete and < 5% contaminated) (Fig. 1e). In total, 107 of the sequenced libraries gave assemblies that appeared to be contaminated based on CheckM results [18], indicating that around 10% of picked colonies may have not in fact been single clones.

The primary point of failure in the workflow appeared to be the liquid culture phase: only 67% of isolates yielded DNA concentrations above 0.1 ng/µL, and 31% above 1 ng/µL (Additional File 1: Fig. S1). Low turbidity of many tubes after incubation was consistent with either slow or no growth in liquid media for many of the colonies transferred from plated media. Initial DNA concentration was a good predictor of subsequent performance: 877 of the 1070 libraries with concentrations ≥ 0.5 ng/µL came from DNA extractions with concentrations above 0.1 ng/µL. Seven hundred four of the 715 high-quality assemblies (98%) came from samples with library concentrations above 0.5 ng/µL (Additional File 1: Fig. S2).

### Isolate diversity and distribution

Of the 715 fully-assembled isolate genomes, 688 were classified successfully with GTDB-Tk [19]. All 688 were classified as Firmicutes, with most (572) belonging to the Bacilli and 162 to the Clostridia. Together, these accounted for 9 unique taxonomic assignments at the order level, 13 at the level of family, and 30 at the genus level; all 688 genomes were assigned to a species. There was a mean of 10.1 unique GTDB taxon strings (standard deviation = 4.2) recovered per host individual. By far the most common genus among the assembled genomes was *Streptococcus* (308), followed by *Enterococcus* “D” group (76), *Staphylococcus* (53), *Clostridium* “P” group (44), and *Blautia* “A” group (42). Taxonomic classifications, assembly statistics, and other metadata for all isolates are presented in Additional File 3.

**Fig. 2:**
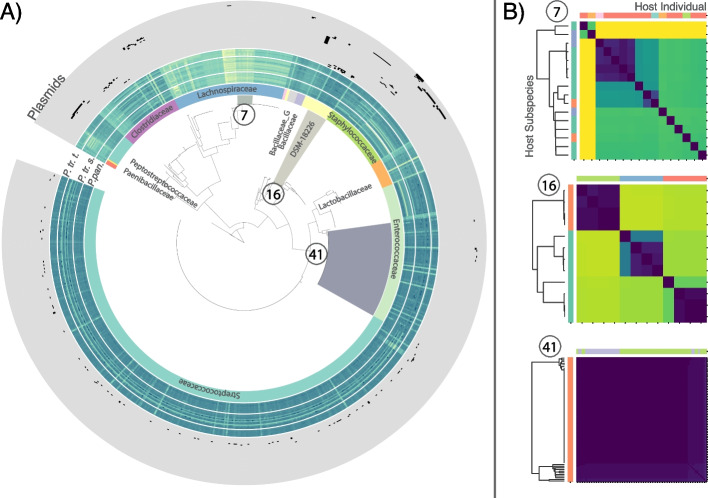
Diversity of bacteria recovered. **a** Multilocus phylogenetic reconstruction from 706 isolate assemblies using Phylophlan and the Amphora2 universal single-copy marker gene set. Colors and labels on the inner ring indicate family-level taxonomic assignment from GTDB-tk. Heatmaps in middle rings indicate log10 estimated coverage per isolate genome from CoverM within metagenomes of wild *P. paniscus*, *P. troglodytes schweinfurthii*, and *Pan troglodytes troglodytes*. Each gray outer ring indicates presence (black) or absence (gray) of a putative plasmid cluster within bacterial isolates. **b** Pairwise Average Nucleotide Diversity among strains within each 95% genome-wide ANI cluster show different patterns of within- “species” diversity revealed by whole-genome screening. Highlighted strains indicated by circled number and colored clade on tree in **a**. Heatmap color values indicate log pairwise nucleotide diversity between each pair of isolates in a cluster. Color bars at the left and annotations at the right show the host species identity of the sample from which the isolate was recovered. Color bars at the top show the host individual

Sourmash, which assigns taxonomy based on kmer composition of reads rather than assemblies, was able to classify more of the isolates, with 828 being classified to at least the phylum level. 827 were classified to order, 812 to family, 797 to genus, and 778 to species level. These classifications were highly consistent with the full-genome taxonomies, with 99% matching at the phylum and class levels, 97% matching at the order and family levels, 97.0% matching at the genus level, and 94% matching at the species level. It should be noted that the phylogenetic placement using marker genes with the GTDB-Tk is likely to give more accurate results, so the additional taxonomic annotations estimated by Sourmash should be considered tentative. Phylogenetic reconstruction using concatenated marker gene sequences was also largely concordant with taxonomic assignment (Fig. 2).

Mapping metagenomic reads sequenced directly from wild chimpanzee and bonobo fecal samples against the assembled isolate genomes supported an origin from those samples. A mean of 2.97% (SD 1.12%) of metagenomic reads mapped to the isolate genome assemblies.

### Isolate population genetics

Assembling individual isolate genomes also allowed us to explore the variation in within-species diversity that might have been hidden by 16S rRNA gene-based screening. Using dRep [20], we clustered the assembled isolates into 45 clusters sharing genome-wide estimated Average Nucleotide Identity (ANI) of > 95%. These clusters of strains correspond roughly to the convention for delineating bacterial species based on ANI divergence. All-by-all ANI comparisons within these clusters indicated differences in within-cluster diversity among clusters (Fig. 2, Additional File 1: Fig. S3). Clusters showed differences in similarity structure ranging from deep divisions with representatives recovered from multiple host individuals and species (e.g., cluster 7, Fig. 2); to clusters with more or less isogenic clones recovered from within individuals but which differed between individuals (e.g., cluster 16, Fig. 2c); to clusters that were entirely clonal, with identical genomes recovered from multiple individuals within the same host species (e.g., cluster 41, Fig. 2d).

A substantial amount of genomic diversity was observed within groups of genomes sharing identical full-length 16S rRNA gene haplotypes. Contigs containing the 16S rRNA gene were recovered from a total of 689 isolate genomes, with 594 genome assemblies containing only a single unique 16S rRNA gene haplotype and 95 containing more than one (see Additional File 3). Among the 39 unique 16S rRNA gene haplotype groups (i.e., groups of genomes sharing the same unique 16S rRNA gene haplotype), the average estimated genome-wide ANI ranged from 100% to 91.6% (mean = 99.15%, StdDev = 1.92%; Additional File 1: Fig. S4).

### Putative plasmid diversity and distribution

One major advantage of bacterial genomes assembled from isolates relative to metagenome-assembled genomes is the ability to confidently associate plasmids with bacterial chromosomes. Here, we leveraged these data to assess the extent to which plasmid communities and sequences have diverged among bacterial lineages and among chimpanzee and bonobo subspecies sampled throughout equatorial Africa (Additional File 1: Fig. S5). We enumerated all plasmids within our isolate assemblies using a recently developed machine learning approach [21] which leverages gene content to predict plasmids. A scan of all isolate assemblies yielded a total of 516 putative plasmid contigs recovered from 258 individual genome assemblies. These belonged to 245 clusters as calculated by MobMess [21], with 64 of these clusters containing more than one contig. Only 31 of these putative plasmids matched existing plasmid sequences in the PLSDB plasmid database [22], indicating that more than 94% of the recovered plasmid sequences were novel.

**Fig. 3:**
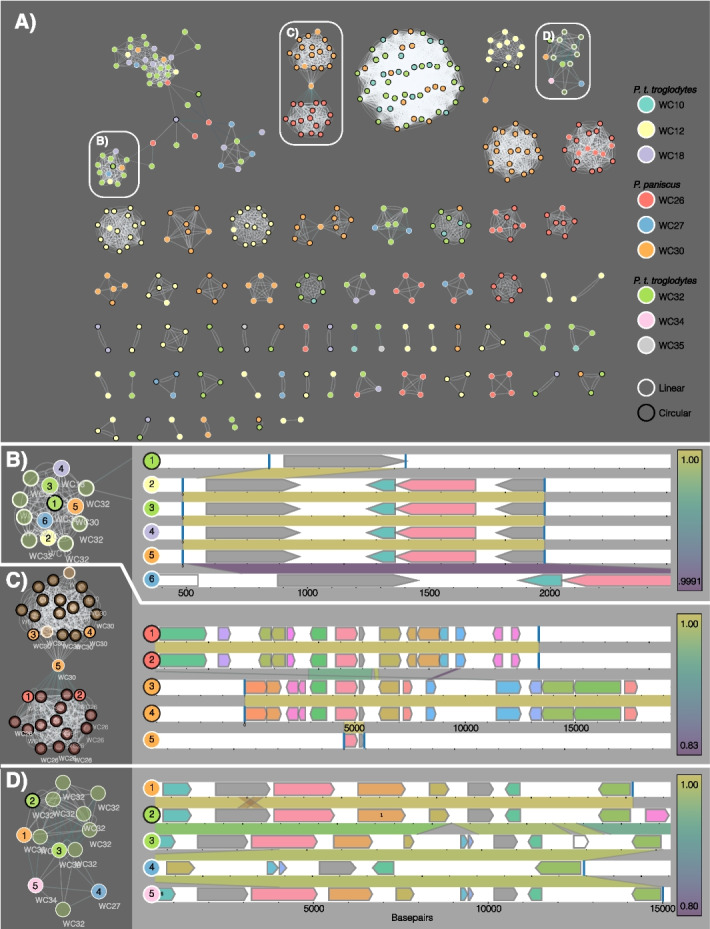
Plasmid sharing among bacteria within and between hosts. **A** Plasmid similarity network as visualized by MobMess. Each network node indicates a putative plasmid predicted from by PlasX, and each edge indicates shared sequence identity with another plasmid. Node fill colors indicate the host ape individual from which the plasmid was sequenced, and node border colors indicate whether the plasmid sequence was determined to be linear or circular. Focal plasmid groups are indicated by a white border. **B** Focal plasmid group with sequence similarity shared among five host individuals. Node colors as in **A**. Right panel shows plasmid predicted gene content, with colored regions between alignments indicating alignment identity between plasmids; alignment identity indicated by the color bar at the right. **C** Focal plasmid group with sequence similarity shared among two *P. paniscus* host individuals. Alignments have higher sequence identity within individuals than between. **D** Focal plasmid group with sequence similarity shared among *P. paniscus* and *P. t. troglodytes* individual. Some plasmids show a high degree of sequence synteny and identity between individuals (e.g., between plasmids 1 and 2, or 3 and 5), while others show sequence insertions and lower identity to related plasmids within the same individual (e.g., between plasmids 2 and 3)

**Fig. 4:**
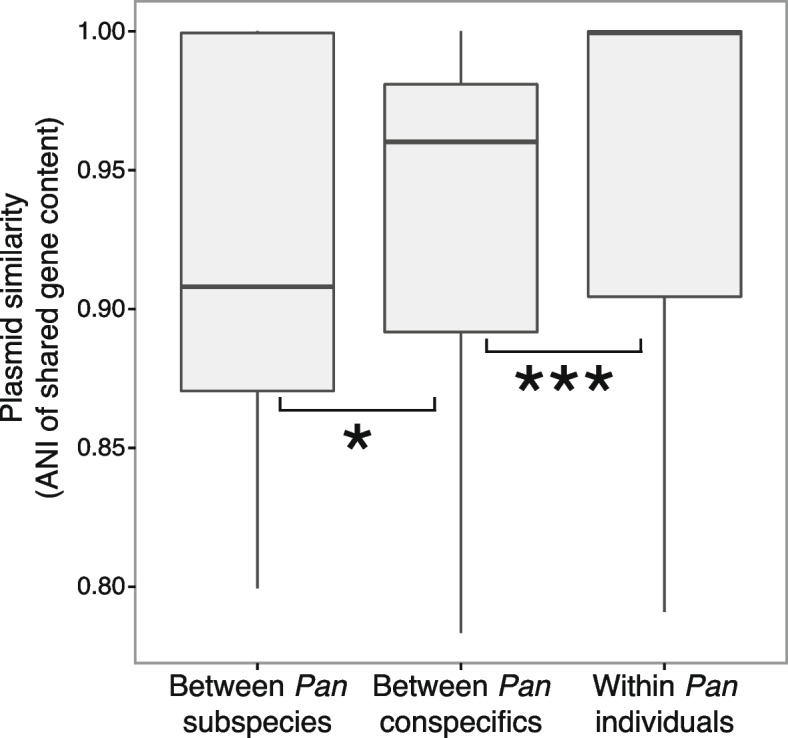
Sequence similarity between homologous regions is higher in plasmids from the same host individual and subspecies. Boxplots show the distribution of average nucleotide identities from local alignments between plasmids recovered from bacteria cultured from different host species (*n* = 6824 edges), different individuals from the same host species (*n* = 2008 edges), or from the same individual (*n* = 5762)

Consistent with phylogenetic and geographic barriers to exchange of plasmids, we found that plasmids were most often shared between genomes of related taxa found within the same bacterial and *Pan* host species (Fig. 3A; Additional File 1: Fig. S6). However, in many cases, plasmids containing homologous stretches of DNA were found in genomes from multiple distantly related bacterial taxa (i.e., different families, Additional File 1: Fig. S6). Similarly, we observed several cases where similar plasmid sequences were detected in multiple *Pan* host individuals (Fig. 3B–D). Across all plasmids, we observed significantly greater nucleotide similarity (i.e., ANI) between homologous regions of plasmids recovered from different *Pan* individuals from the same *Pan* species than between homologous regions of plasmids recovered from different *Pan* individuals from different *Pan* species (Fig. 4; Mann–Whitney *U* test *p*-value = 0.036). Nucleotide similarity was greater still among homologous regions of different plasmids recovered from the same host individual (Fig. 4; Mann–Whitney *U* test *p*-value < 0.0001). These results indicate the divergence of homologous plasmid sequences between *Pan* host species.

### Protocol cost estimates

Costs are difficult to estimate and communicate accurately, as purchasing prices and available equipment vary widely among laboratories. However, as one of the primary motivations of this manuscript is to make high-throughput isolate genome sequencing accessible to as many researchers as possible, we give our best estimates for both our required capital investment and per-sample consumable costs (Additional File 2: Table S1) as a point of reference.

Our laboratory already had basic molecular biology equipment, including PCR machines, centrifuges, manual pipettes, and access to a fluorescence plate reader and bead beater. Additional capital expenses required for this protocol included an OpenTrons OT-2 robot with 2 multi-channel pipettes and a magnetic plate expansion module, a strip tube bead beater adapter, and materials costs for the 3D-printed labware; in total, capital expenses amounted to approximately $13,000.

We estimate a per-sample consumables cost of around $10. Of this, liquid culture and DNA extraction account for about $1.50, library preparation around $3, and sequencing around $5. Even with the 36% success rate we observed here, with no culling of failed samples prior to sequencing, this equates to around $25 per high-quality genome assembly.

## Discussion

We developed a workflow for high-throughput bacterial genome sequencing from complex microbiota. Our workflow makes use of custom-designed 3D-printed labware, the relatively inexpensive OpenTrons liquid handling platform, and recently developed methods for Illumina library preparation using highly diluted reagents. Together, this combination of methods allowed the library preparation and whole-genome sequencing of hundreds of bacterial isolates in parallel for a marginal cost of ~ $10 per isolate. Importantly, by reducing per-isolate whole-genome sequencing costs substantially, our workflow alleviates the need for 16S rRNA gene- or mass spectrometry-based approaches for dereplicating bacterial strains prior to whole-genome sequencing. Of the bacterial isolates that grew in liquid culture and yielded appreciable DNA concentrations post-extraction (i.e., “Pass” in Columns 1 and 2 in Fig. 1g), > 80% yielded Hackflex libraries, nearly all of which yielded genome drafts upon sequencing (Fig. 1). Metagenomic data can be used to assemble contiguous sequences within bacterial chromosomes and plasmids, but these data alone struggle to capture fine-scale strain-level diversity and cannot fully determine the distributions of chromosomes and plasmids among bacterial cells. In contrast, whole-genome sequencing of bacterial isolates affords the opportunity to definitively associate plasmids with their bacterial hosts. We demonstrate the utility of this approach by isolating and profiling strain-level bacterial diversity in gut microbiota of wild chimpanzees and bonobos.

Machine-learning classification of assemblies discovered hundreds of previously undescribed plasmids in chimpanzee and bonobo gut bacterial isolates. Analyses of plasmid distributions among bacterial and *Pan* hosts revealed plasmids shared between distantly related bacterial lineages both within and between *Pan* individuals (Additional File 1: Fig. S6), consistent with horizontal gene transfer (HGT) within microbiota [13, 23, 24]. Although there were some observed instances of related plasmids being shared between bacterial taxa, the limited sample size of host individuals and restricted window into overall bacterial diversity limits our ability to make broad inferences about distribution. Interestingly, though, many homologous sequences were also shared between *Pan*-host species (Fig. 3). Of these sequences, sequence divergence between *Pan* species was significantly higher than that between conspecific *Pan* individuals (Fig. 4). These results indicate divergence of gut bacterial plasmid sequences between primate-host species lineages. Thus, isolate sequencing enables chromosome- and plasmid-resolved genomic analyses of bacterial species that remain difficult with metagenomic data alone.

Our workflow has several advantages and disadvantages relative to existing approaches for high-throughput bacterial isolation and whole-genome sequencing. One major advantage is its simplicity, as it relies on standard microbiological and molecular biology approaches and is fully automated on the OpenTrons platform. For example, relative to microfluidics-based isolation [25, 26] or single-cell genome sequencing approaches [27, 28], our method is readily applicable by labs without the need for capital-intensive specialized equipment. The equipment costs necessary to execute our full protocol are also dramatically lower than for a number of previously-developed high-throughput genome sequencing workflows that achieve low marginal costs using expensive robotics [16, 17, 29]. Similarly, while Hi-C-based approaches also have the ability to link plasmids with their host bacteria, these methods rely on labor-intensive protocols that crosslink chromatin with formaldehyde, then digested, and re-ligated to isolate covalently linked DNA fragments [27]. Moreover, both droplet and Hi-C approaches typically capture only a fraction of the genome, and they in general do not allow for the retention of isolated cultures for further experimental study. In contrast, a weakness relative to single-cell and Hi-C approaches is that our workflow can only interrogate bacteria that can be cultured and isolated.

The data we report here represent the first two complete full-scale sequencing runs from this protocol, and there are still opportunities for improvement. First, the loss rate could likely be improved through further optimization of pipette accuracy and precision. We also note that approximately one third of bacterial colonies grown in isolation failed to yield appreciable concentrations of DNA (> 0.1 ng/uL), a failure rate that could likely be reduced by screening for isolate growth prior to extraction. Similarly, the rate of contamination (or sequence libraries containing DNA from multiple bacterial types) could be reduced by adding a secondary re-streaked plate culture step rather than picking directly into liquid culture; in this experiment, we chose to pick directly to maximize throughput. Second, although the protocols we provide can in principle be run with very little specific prior training or programming experience, some working knowledge of Python programming in general, and the Opentrons Python API in particular, is helpful. And third, the logistical challenges of moving from hundreds to potentially tens of thousands of samples—including storage, labeling, and in particular sample provenance validation and metadata tracking—are largely unaddressed here. We will be continuing to address each of these issues in future development of these protocols.

To ensure the greatest utility of our workflow for the research community, all protocols and hardware schematics are freely available for public use at https://github.com/tanaes/Moeller_Opentrons_protocol_library [30], https://github.com/tanaes/opentrons_functions [31], and https://github.com/CUMoellerLab/Labware [32]. These repositories will be maintained and updated as we make further additions and improvements to the protocols in the future.

The isolates sequenced in this study represent, to our knowledge, the first large-scale compendium of cultured bacterial genomes from wild chimpanzee and bonobo gut microbiomes. The samples used to demonstrate the methods in this paper were far from ideal for purposes of generic cultivation: preserved in RNALater, and stored for many years at − 80 °C, a substantial portion of the diversity in the original samples was most likely no longer viable, resulting in a dearth of some common gut bacterial lineages in our dataset (Fig. 2). Although the entire process of cultivation, from initial inoculation of plates through colony isolation and regrowth in liquid media took place within an anaerobic chamber, we did not recover many expected anaerobes, leading to uncertainty of whether the preservation or growth conditions may have been responsible for their absence. Accordingly, only 3% of the metagenomic diversity detected by sequencing of the fecal samples used for cultivation was present in our dataset of isolate genomes. Fresh samples (or those collected into cryoprotectants specifically for purposes of later cultivation) will no doubt yield a greater diversity of original gut cultivars. However, the genome resources generated from this wild hominid gut bacterial isolate collection complement and enable comparative analyses with existing gut bacterial genome databases for a subset of chimpanzee and bonobo gut bacterial taxa. All isolates generated by this study have been preserved in glycerol stocks and are available upon request for research purposes.

## Conclusions

The vast majority of global microbial genomic diversity remains unexplored. While centralized efforts to explore microbial diversity of particular significance to human health are generating enormous amounts of new data, exploration of most other environments most often occurs in a more decentralized fashion, often by researchers with less access to the capital equipment and economies of scale enjoyed by their medically-oriented peers. Many rare or endangered host species are represented in existing collections, often by samples collected for purposes other than microbial cultivation. Such collections represent a potentially vast resource for exploring naturally occurring host-associated microbes if cost-effective methods exist to access them. The protocols presented here expand the accessibility of high-throughput microbial genomics, thereby increasing the diversity of environments from which microbial isolates and reference genomes can be obtained. Given the interconnectedness of microbial genomic diversity in nature, expanding the breadth of such data will be of substantial benefit to researchers studying microbes from all sorts of environments.

## Methods

To accomplish our goals of maximum isolate genome throughput with minimal capital and labor costs, we developed a workflow based around the OpenTrons OT-2 robotic liquid handling platform (Fig. 1). This instrument allows for repeatable automation of many protocols, while costing less than $10,000 as configured. Where possible, we took advantage of previously-published low-cost molecular biology protocols, adapting them for automation on the OpenTrons platform. All the protocols described here are available at https://github.com/tanaes/Moeller_Opentrons_protocol_library [30]. In addition, we wrote extensions to the OpenTrons Protocol API to improve certain aspects of instrument behavior, especially relating to use with magnetic bead protocols. An installable library of these extensions is available at https://github.com/tanaes/opentrons_functions [31].

For some protocols, we found that there were key steps that would require laboratory apparatus that were either not available for commercial purchase, uncommon in a typical molecular biology lab, or would require substantial investment. For these steps, we designed our own versions suitable for rapid manufacture with 3D printers and/or laser cutters and using inexpensive commodity electronic components. These apparatus were designed using Fusion360 CAD software (Autodesk, Inc.). Full source files and component lists can be found at https://github.com/CUMoellerLab/Labware [32]. All data in this paper were generated using versions of the 3D-printed apparatus described below, rather than commercially purchased alternatives. Commercial alternatives to all lab-built equipment are listed in Additional File 2: Table S2.

### Sample collection, storage, and metagenome sequencing

To develop our high-throughput sequencing methodology, we used a set of 10 fecal specimens collected from wild chimpanzees and bonobos throughout equatorial Africa (Additional File 1: Fig. S5) between July 2003 and August 2014 (Additional File 2: Table S3). Samples were collected in the field at *Pan* nest sites, preserved and shipped in RNALater at room temperature, then frozen at − 80 °C for long-term storage [33–39]. These samples were expected to yield only a limited taxonomic fraction of the original microbial population: preservation in RNALater renders many types of microorganisms nonviable. However, our previous work has shown that some portions of the native microbiome from mammalian fecal samples remain viable even after long-term storage in this preservative [16], thereby allowing cultivation of a subset of the original community. Thus, this harsh selective filter against most bacteria was expected to facilitate the recovery of a common subset of gut bacterial species from multiple chimpanzee and bonobo hosts.

For metagenome sequencing, samples were centrifuged and approximately 50 mg of material removed from the pellet for DNA extraction. We extracted metagenomic DNA from pellets using the Qiagen PowerSoil extraction kit. Libraries were generated from metagenomic DNA using the “Illumina Equivalent” library prep method at the Cornell Biotechnology Research Center, and pooled libraries sequenced using an Illumina NovaSeq instrument at the UC Davis Sequencing Center.

### Cultivation

Ten fecal samples—three from *P. paniscus*, three from *P. t. troglodytes*, and four from *P. t. schweinfurthii*—were selected for cultivation. To capture as much of the bacterial diversity that remained viable in RNAlater as possible, we used several different media for cultivation: Yeast Casitone Fatty Acids (YCFA), YCFA + Starch, *Bifidobacterium* selective media (BSM), Brain heart infusion-supplemented (BHIS), and *Bacteroides* Bile Esculin (BBE) (Additional File 2: Table S4). Recipes for all media were derived from [33]. For each sample-by-medium combination, 100 μl of fecal material suspended in RNAlater was plated in an anaerobic chamber (Coy brand) on solid media. Plates were inoculated and incubated at 37 °C for five days in an anaerobic (5% hydrogen, 5% carbon dioxide, and 90% nitrogen) chamber (Coy Lab Products Inc). During each round of cultivation, blank control plates were kept in the anaerobic chamber along with the swabbed experimental plates to check for unintentional environmental contamination.

Liquid culture of picked colonies represented a potential throughput bottleneck, especially if isolates were cultured in conventional glass test tubes. To increase throughput, we instead grew colonies in 1.2 mL 96-place strip tube racks, which have the footprint and well spacing necessary for processing on the OpenTrons liquid handler. Individual colonies were picked by hand from plates into 900 µL of liquid media (Additional File 2: Table S4) using a sterile wooden toothpick without removal from the anaerobic chamber. Then, plates were incubated at 37 °C in the anaerobic chamber for 4 days.

To improve growth in liquid culture for cells that might benefit from increased waste gas diffusion or nutrient distribution, we designed small single-plate orbital shakers to fit inside our anaerobic incubator (Fig. 1b). Adapting an existing open-source design (https://learn.adafruit.com/crickit-lab-shaker/3d-printing), we simplified the electronic components, relocated all connections and controls to the front of the apparatus to facilitate use within the incubator, and changed it to use 5 V USB input for power, allowing us to use a single USB charger to power 7 individual shakers within the incubator.

Following anaerobic incubation, plates were removed from the chamber and 300 µL of media per tube was transferred to a clean deep-well plate and cells pelleted in a centrifuge at 16,000 g. After removal of supernatant, cells were resuspended in glycerol buffer and stored at -80 °C for future use.

### DNA extraction

Kit-based DNA extraction protocols typically cost between $3 and $5 per sample. For 16S rRNA gene amplicon-based screening, this step can sometimes be omitted with a chemical lysis prior to amplification. For whole-genome screening, we judged that the added complexity of a DNA extraction step was necessary. To reduce costs, we adapted the magnetic bead-based extraction methodology from Oberacker et al. [40], which uses laboratory-made reagents and either purchased or lab-made magnetic beads, for use on the OpenTrons platform.

For cell lysis, we chose to use beadbeating to ensure lysis of a broad range of bacterial cell types. We designed a 3D-printed and laser-cut loading system to precisely load 0.2 mm glass beads directly into the 96-well strip-tube plates (Fig. 1b) after pelleting cells and removing liquid media. After bead loading, 800 µL of guanidine HCL lysis buffer was added to the tubes, and they were capped and shaken on an Omni Bead Ruptor Elite at 6.5 m/s for 40 s. The tubes were then spun down on a centrifuge at 400 × *g* for 5 min, decapped, and then moved to the OpenTrons instrument for the remainder of the extraction. The detailed OpenTrons extraction protocol can be found in the project repository linked above. Briefly, the robot transfers 600 µL of lysate to a new plate, adds magnetic beads in a PEG-based binding buffer, and then goes through a series of magnetic binding and wash steps before eluting the extracted DNA in nuclease-free water.

We found extraction efficiency was greatly improved by gently agitating magnetic beads during the initial binding step. To accomplish this, we designed a 3D-printed rotator (Fig. 1c) with attachments for holding 96-well plates or microcentrifuge tubes. After transferring lysate and adding beads and binding buffer on the liquid handler, we programmed a pause to allow the user to remove the plate, seal it, and place it on the rotator for 10 min. Following this step, the plate was unsealed and returned to the liquid handler for the remainder of the protocol.

Extracted DNA was quantified in 384-well plates using a reduced-volume version of the QuantiFluor (Promega) fluorescence-based assay. Four 96-well plates (each the output from a single extraction protocol) were tested in each assay, using an OpenTrons protocol for sample transfer and a Tecan Infinite M200 plate reader for quantification.

### Library prep and sequencing

To inexpensively generate sequencing libraries from thousands of DNA extractions, we adapted the Hackflex library prep protocol [41] to the OpenTrons liquid handler. Briefly, this protocol dilutes key reagents from the Illumina Library Prep protocol to stretch a single kit across more samples. Our adaptation of the protocol changes some reagent quantities to better fit the constraints of the OpenTrons format; for details, see the full protocol in the project repository linked above.

For the libraries presented here, we used barcoded library amplification primers purchased from the Cornell Biotechnology Resource Center. Initially, these shared a single i5 index per library plate, with unique i7 primers per sample. For later libraries, we switched to unique dual indexed (UDI) primers, with 96 unique i5 and i7 primers per plate. To facilitate multiplexing across library prep plates with UDIs, we created a version of the protocol to cycle column matches between i5 and i7 primer plates, allowing up to 12 library plates to be multiplexed without repeating an index combination. Libraries were amplified using 17 cycles of PCR prior to bead-based dual-sided size selection and final elution.

Final libraries were quantified by QuantiFluor (Promega) in 96 well plates, then pooled according to the following algorithm: the volume necessary to transfer 5 ng of library DNA was calculated; for samples requiring more than 10 µL to reach 5 ng transferred (likely failed libraries), 1 µL was transferred; for samples requiring less than 0.5 µL, 0.5 µL was transferred. Per-plate pools were combined and concentrated using magnetic beads and then provided to the Cornell Biotechnology Resource Center for sequencing on an Illumina NextSeq 500 instrument. Two separate sequence runs were performed, combining 13 and 10 library prep plates, respectively.

### Sequence analysis

Isolate genomes derived from fecal samples preserved in RNAlater, which selects for a subset of the bacteria in the *Pan* microbiota [33], enabled analyses of intraspecific bacterial genomic diversity within and among *Pan* individuals and populations. To demonstrate the utility of isolate genomes for strain-level analyses of microbiota, we focused on two sets of analyses of genomic diversity that remain difficult or not possible with shotgun metagenomic data alone.

First, we characterized intraspecific bacterial genomic diversity both within and between individual *Pan* hosts. Isolate sequences were processed using the Bactopia pipeline [42]. This pipeline does sequence trimming and QC with FastQC (https://www.bioinformatics.babraham.ac.uk/projects/fastqc/), assembled sequences with Shovill (https://github.com/tseemann/shovill) and SKESA [43], and performs assembly quality checking with CheckM [18]. Gene prediction and annotation was performed with Prokka [44]. Taxonomy of each isolate sequence library was estimated from unassembled reads using Sourmash with the GTDB R06-RS202 LCA-formatted database and k = 31 [45]. To create a phylogeny of isolates, assembled genomes predicted to be less than 5% contaminated with CheckM were processed using PhyloPhlAn2 [46] using the Amphora2 marker set [47] and the “Fast / High Diversity” default settings. To estimate the relative abundances of isolates in original samples, we used CoverM (https://github.com/wwood/CoverM) to calculate coverage for each isolate genome in each of the available chimpanzee metagenomes. 16S rRNA gene sequences were recovered from isolate genome assemblies using PhyloFlash [48] as implemented in Bactopia. To identify unique haplotypes, the complete alignment of all 16S rRNA gene sequences from PhyloFlash was manually inspected for positions corresponding to the commonly-used 27F and 1492R primers and trimmed to just the portion internal to those priming sites.

Second, we identified plasmids within each isolate genome assembly to assess the distribution of and similarity of these mobile elements among bacterial and chimpanzee hosts. Putative plasmid contigs were identified using PlasX [21], which uses a machine learning algorithm to classify mobile elements based on their gene content. Open reading frames from contigs were annotated in Anvi’o [49] and supplied to the PlasX algorithm. Contigs scored 0.90 or higher by PlasX were considered putative plasmids. Circularity of putative plasmids was assessed based on read mapping according to the algorithm used by Yu, Fogarty, and Eren [21]. To assess novelty relative to current known plasmid sequences, putative plasmid sequences were searched against the latest version of the PLSDB plasmid database (v. 2021_06_23_v2, [22]) using BLASTn [50], and considered as previously observed if they matched at least one database sequence across at least 90% of the query length at ≥ 60% sequence identity. Finally, putative plasmid similarity networks and gene alignment visualizations were generated using MobMess [21].

## Supplementary Information

Additional file 1: Figure S1. Alluvial plot of protocol efficiency. Figure S2. Relationship between assembly quality and library concentration. Figure S3. Intraspecific nucleotide diversity. Figure S4. Genomic dissimilarity within 16S haplotypes.

Additional file 2: Table S1. Equipment sourcing and alternatives. Table S2. Sample information. Table S3. Culturing information. Table S4. Cost estimates.

Additional file 3. Isolate taxonomic information, genome assembly statistics, and other metadata.

Additional file 4. Review History.

## Data Availability

All raw sequence data from this publication are available in the Qiita data repository, study number 14410 (https://qiita.ucsd.edu/study/description/14410), as well as at the EBI ENA repository with accession number ERP136830 [51]. Opentrons protocols are available at https://github.com/CUMoellerLab/Moeller_Opentrons_protocol_library [30] and custom function library at https://github.com/CUMoellerLab/opentrons_functions [31]. Printable labware files and assembly instructions are available at https://github.com/CUMoellerLab/Labware [32]. Isolates are available upon request.
