# Supplementary material for: A low-cost genomics workflow enables isolate screening and strain-level analyses within microbiomes
Source: Genome Biol. 2022 Oct 12;23:212. doi: 10.1186/s13059-022-02777-w (PMC9558970; doi:10.1186/s13059-022-02777-w)
Supplement: Supplementary file 1 — Additional file 1: Figure S1. Alluvial plot of protocol efficiency. Figure S2. Relationship between assembly quality and library concentration. Figure S3. Intraspecific nucleotide diversity. Figure S4. Genomic dissimilarity within 16S haplotypes. [file 13059_2022_2777_MOESM1_ESM.pdf]

Supplementary Information

Supplementary Figures

Figure S1. Alluvial plot of protocol efficiency.

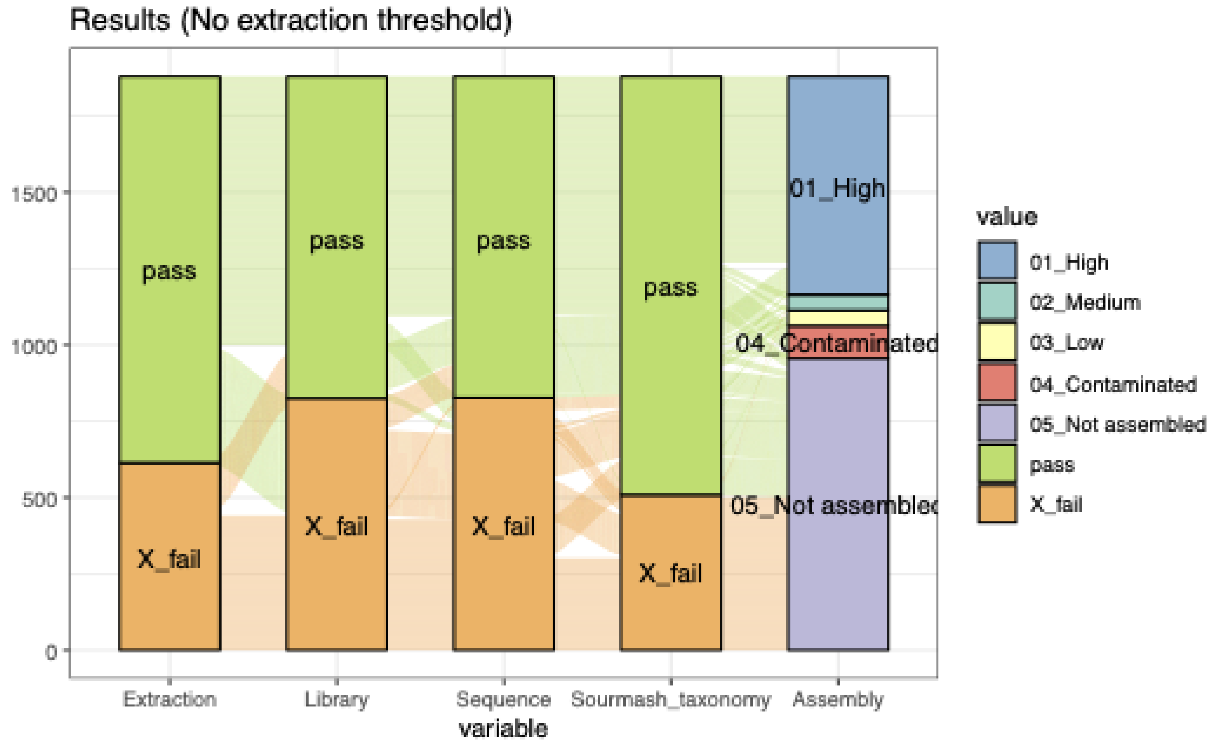

Figure S1. Alluvial plot of protocol efficiency. Results from initial rounds of screening, showing samples passing certain QC thresholds at each stage (extraction: 0.1 ng/µL DNA concentration; Library prep: 0.5 ng/µL DNA concentration; Sequencing: 25 Mbp sequence yield; Taxonomy: taxonomy assigned by Sourmash; Assembly: High, Medium, and Low-quality assemblies), Contaminated assemblies, and unassembled samples. Colored lines connect the same sample through each stage of the chart.

Figure S2. Relationship between assembly quality and library concentration.

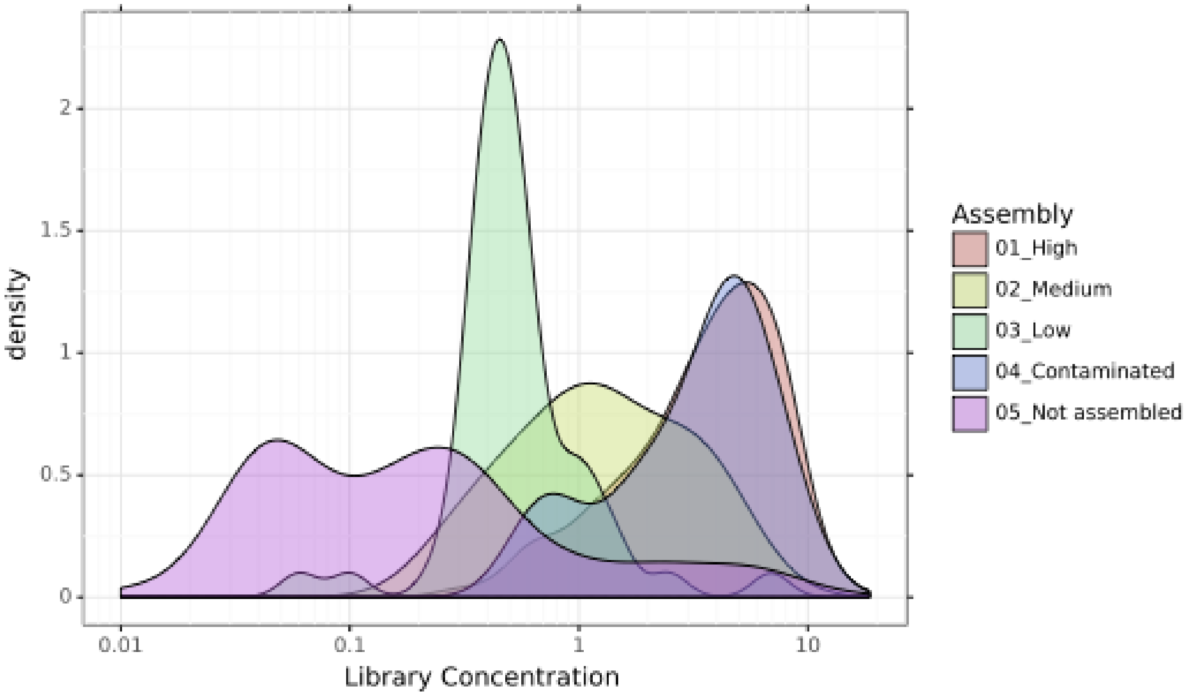

Figure S2. Relationship between assembly quality and library concentration. Kernel density plots showing distribution of sequence library DNA concentrations for each level of assembly quality. As expected, assembly quality generally increases with library concentration, with libraries above 1 ng/µL typically yielding medium or high quality assemblies. Contaminated assemblies had library concentrations very similar to "high quality" uncontaminated libraries, suggesting contamination from multiple inocula rather than low-concentration reagent contamination.

Figure S3. Intraspecific nucleotide diversity.

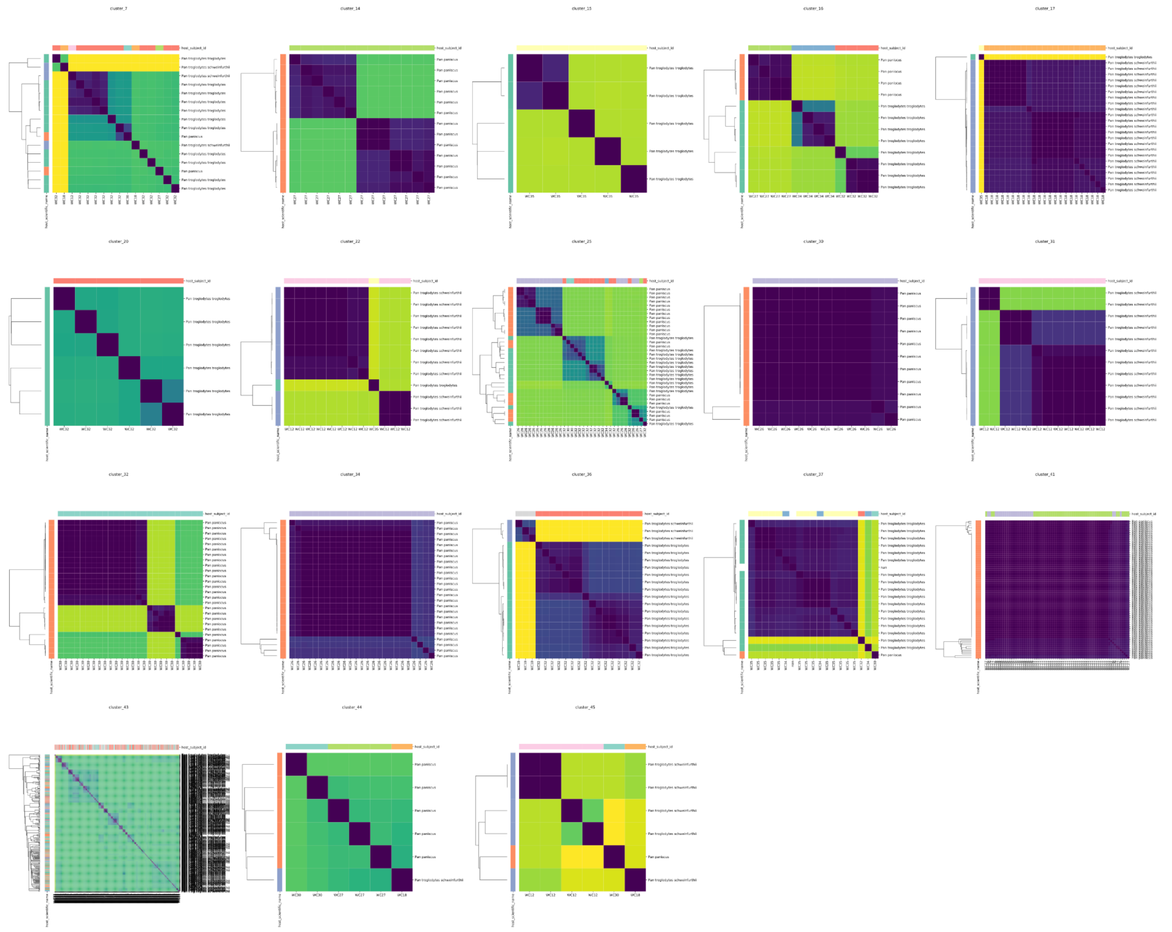

Figure S3. Intraspecific nucleotide diversity. Pairwise Average Nucleotide Diversity among strains within each cluster show different patterns of within-‘species’ diversity revealed by whole-genome screening. Heatmap color values indicate log pairwise nucleotide diversity between each pair of isolates in a cluster. Color bars at left and annotations at right show host species identity of the sample from which the isolate was recovered. Color bars at top show the host individual. Different patterns highlight differences in the distribution and quantity of nucleotide variation in different isolate clusters, ranging from nearly clonal isolates recovered from within a single (clusters 30 and 34) or across multiple (cluster 41) individuals; to moderate amounts of variation largely partitioned across host individuals (cluster 16); to highly structured variation possibly indicative of multiple ‘species’ grouping within the same 95% ANI threshold (cluster 7 and 36).

Figure S4. Genomic dissimilarity within 16S haplotypes

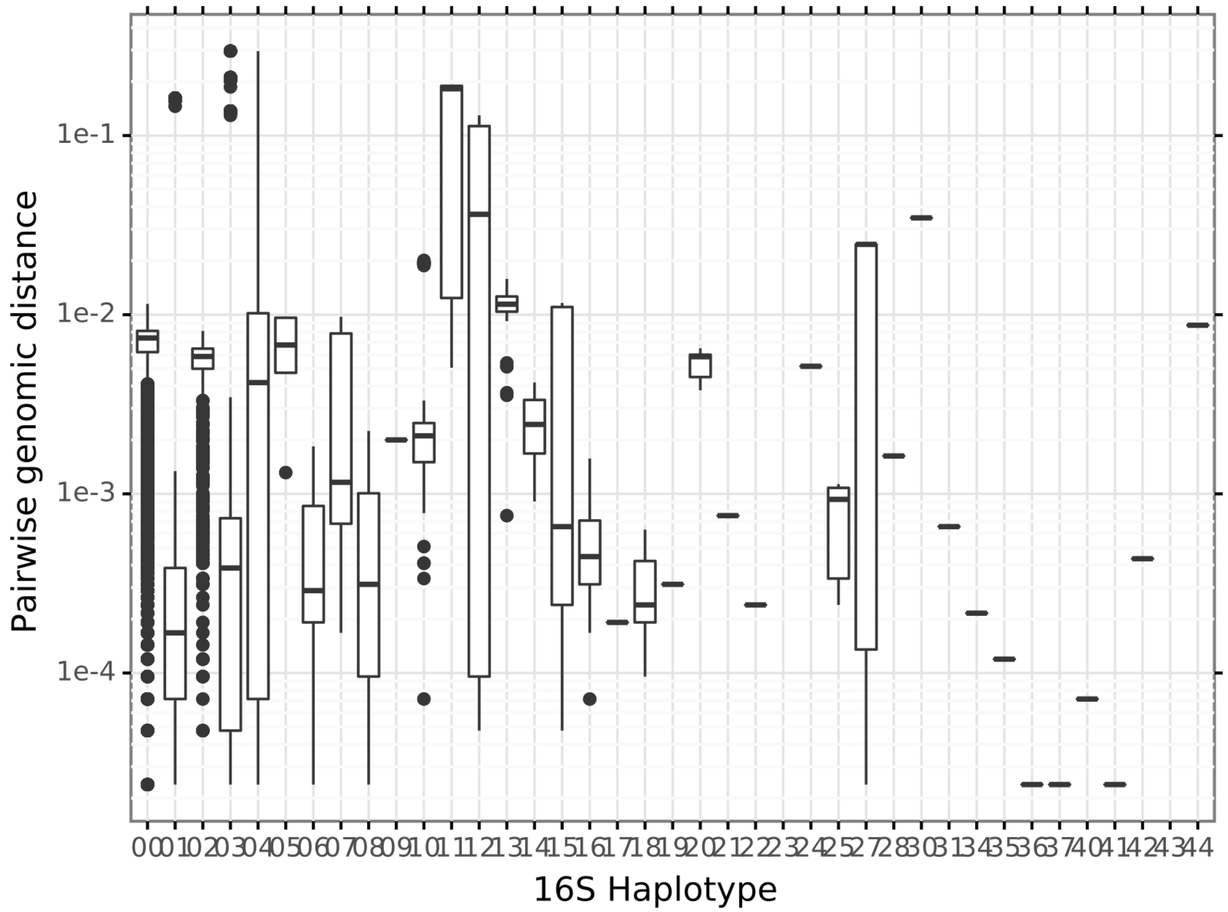

Figure S4. Genomic dissimilarity within 16S haplotypes. Each boxplot indicates the estimated genome-wide dissimilarities between all pairs of genomes containing single, identical 16S rRNA gene haplotypes.

Figure S5. Map of sample collection locations

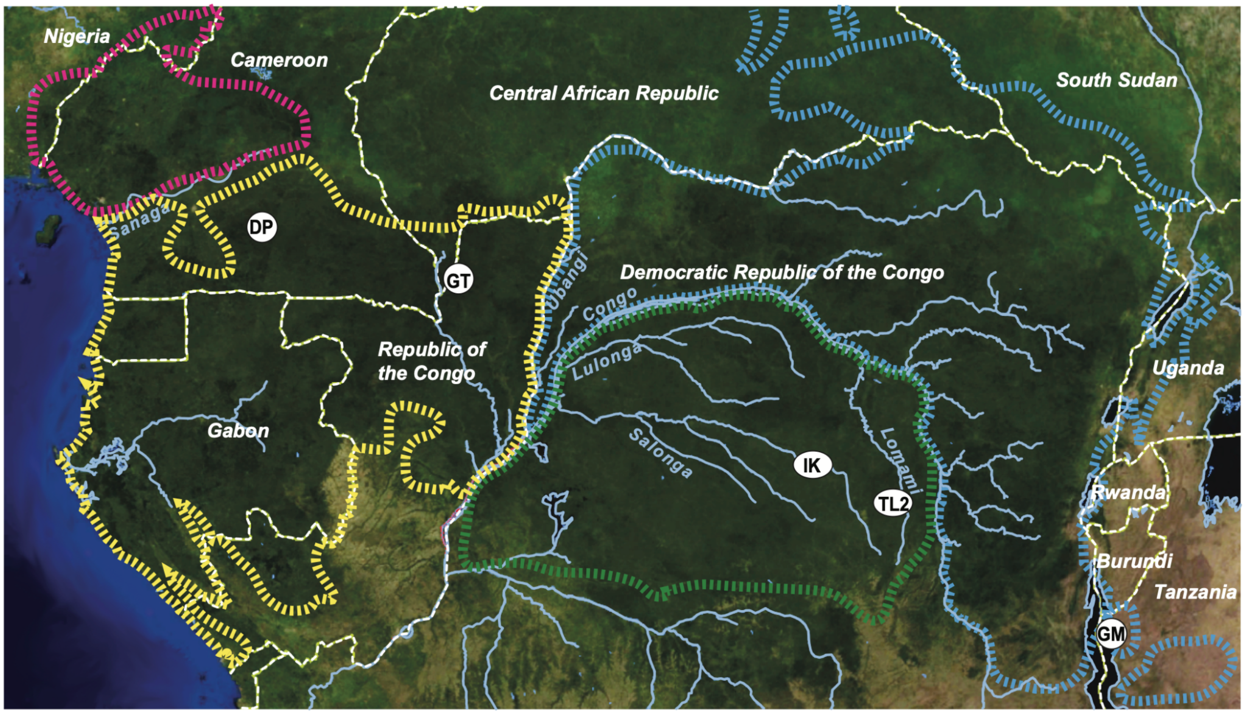

Figure S5. Map of sample collection locations. Two-letter site codes correspond to the first two letters of sample names in Table S2.

Figure S6. Plasmid similarity network colored by bacterial family

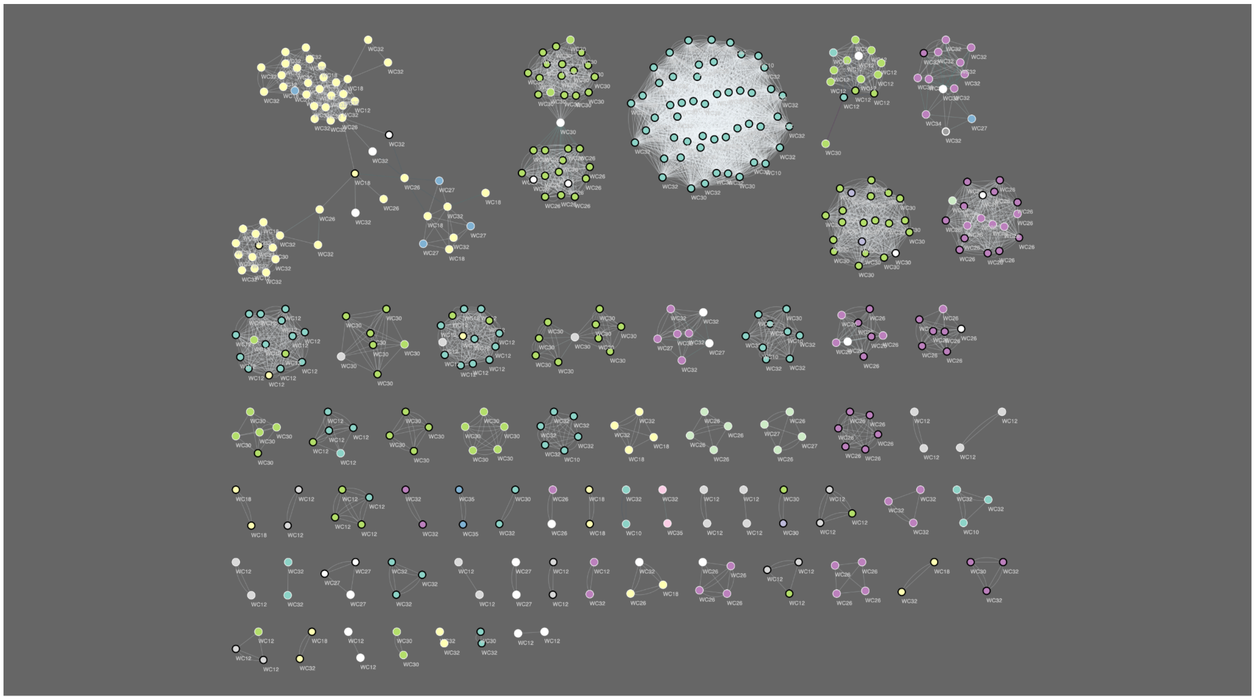

Figure S6. Plasmid similarity network colored by bacterial family. Network is topologically identical to that in Figure 3A, but with nodes colored by family-level taxonomy of bacterial host. Taxonomy colors same as in Figure 2.
