# Supplementary material for: A low-cost genomics workflow enables isolate screening and strain-level analyses within microbiomes
Source: Genome Biol. 2022 Oct 12;23:212. doi: 10.1186/s13059-022-02777-w (PMC9558970; doi:10.1186/s13059-022-02777-w)
Supplement: Supplementary file 2 — Additional file 2: Table S1. Equipment sourcing and alternatives. Table S2. Sample information. Table S3. Culturing information. Table S4. Cost estimates. [file 13059_2022_2777_MOESM2_ESM.pdf]

Supplementary Information

Supplementary Tables

Table S1: Equipment sourcing and alternatives

Apparatus	Instructions	Use	Approximate Materials Cost	Commercial Alternative	Commerical Alternative Link	Commercial Alternative Cost
Plate shaker	https://github.com/CUMoellerLab/Labware/tree/main/Plate_shaker	Shaking plates during liquid culture	$35.00	Boekel Scientific Shaker	https://us.vwr.com/store/product/13921948/microplate-shakers-boekel	$1333.00
Bead dispenser	https://github.com/CUMoellerLab/Labware/tree/main/Bead_dispenser	Loading lysis beads into strip tubes	$85.00	LabTie Gravity Bead Loader	https://biospec.com/products/labtie-bead-dispensers?taxon_id=29	$3626.00
Plate/Tube rotator	https://github.com/CUMoellerLab/Labware/tree/main/Rotator	Keeping magnetic beads suspended during extractions	$45.00	RotoFlex Plus Tube Rotator with plate adapter	https://us.vwr.com/store/product/10148591/accessories. https://us.vwr.com/store/product/7928492/rotoflex-tube-rotator-argos-technologies	$1768.00

Table S2: Sample information

**Individual**	**GM_Code**	**Collection_Date**	**Name**	**Sex**	**Subspecies**	**Site**
WC02	GM0566	2004-08-30	Ch-095	*F*	*Pan troglodytes schweinfurthii*	KL
WC10	GM3878	2014-08-17	Kati (Tita)	*F*	*Pan troglodytes schweinfurthii*	KL
WC12	GM3909	2014-08-27	Kazi	*M*	*Pan troglodytes schweinfurthii*	KL
WC18	GM3902	2014-08-26	Pairotti_Poa	*F*	*Pan troglodytes schweinfurthii*	KL
WC26	KSG2915	2010-05-18	IK2915		*Pan paniscus*	Ikela, Bafeke-Balanga
WC27	KSG3821	2012-11-19	TL3821		*Pan paniscus*	Tshuapa-Lomami-Lualaba
WC30	KSG3845	2012-11-26	TL3845		*Pan paniscus*	Tshuapa-Lomami-Lualaba
WC32	DP130	2003-11-07	MP130		*Pan troglodytes troglodytes*	Cameroon, Doumo Pierre
WC34	DP095	2003-07-29	MP95		*Pan troglodytes troglodytes*	Cameroon, Doumo Pierre
WC35	GT504	2005-02-22			*Pan troglodytes troglodytes*	RC, Goualougo Triangle

Table S3: Culturing information

Plate	Host	Culture date	Culture medium
WC10-BSM-1	WC10	2020-2-17	BSM
YS12	WC12	2020-2-27	YCFA+Starch
BBE18	WC18	2020-2-27	BBE
BSM18-1	WC18	2020-2-27	BSM
BSM18-2	WC18	2020-2-27	BSM
YS18	WC18	2020-2-27	YCFA+Starch
YS-26-1	WC26	2020-2-20	YCFA+Starch
YS-26-2	WC26	2020-2-20	YCFA+Starch
27-YS-1	WC27	2020-2-20	YCFA+Starch
27-YS-2	WC27	2020-2-20	YCFA+Starch
BHIS-30	WC30	2020-2-17	BHIS
WC-30	WC30	2020-2-17	YCFA
YS-30-3	WC30	2020-2-17	YCFA+Starch
YS-30	WC30	2020-2-17	YCFA+Starch
32-YCFA-2	WC32	2020-2-17	YCFA
BHIS-32	WC32	2020-2-17	BHIS
BSM-32	WC32	2020-2-17	BSM
WC-32	WC32	2020-2-17	BHIS
WC32-BSM-2	WC32	2020-2-17	BSM
BBE-34-35	WC34	2020-2-24	BBE
YS-35-1	WC35	2020-2-24	YCFA+Starch
YS-35-2	WC35	not recorded	YCFA+Starch

Table S4: Cost estimates

Item	Step	Qty used	Stock price	Stock qty	Stock unit	Net cost
96 well strip plate	Extraction	1	$269.90	50	ea	$5.40
GITC buffer	Extraction	40	$28.30	1000	mL	$1.13
Magnetic beads	Extraction	2.5	$223.00	24	mL	$23.23
Isopropanol	Extraction	30	$19.05	4000	mL	$0.14
Ethanol	Extraction	60	$25.00	4000	mL	$0.38
VWR deep well plate	Extraction	1	$187.85	50	ea	$3.76
Bio-Rad PCR plate	Extraction	1	$1,366.40	400	ea	$3.42
OpenTrons 300 µL tips	Extraction	2	$3,025.00	1000	ea	$6.05
OpenTrons 200µL filter tips	Extraction	2	$687.50	100	ea	$13.75
glass lysis beads	Extraction	48	$40.00	453	g	$4.24
					Subtotal:	$61.49
Illumina Library Prep Kit	Library Prep	96	$1038.00	960	rxn	$103.80
Primestar GXL Polymerase	Library Prep	225	$671.00	1000	units	$150.98
Index primers	Library Prep	1	$500.00	16	plates	$31.25
SPRI beads	Library Prep	6	$3,835.80	500	mL	$46.03
Tagmentation buffer	Library Prep	3.2	$40.34	1000	mL	$0.13
Stop buffer	Library Prep	1.6	$0.86	1000	mL	$0.00
Wash buffer	Library Prep	24	$8.14	1000	mL	$0.20
Nuclease free H2O	Library Prep	8	$30.22	1000	mL	$0.24
Ethanol	Library Prep	36	$25.00	4000	mL	$0.23
Bio-Rad PCR plate	Library Prep	3	$1,366.40	400	ea	$10.25
OpenTrons 300 µL tips	Library Prep	1	$3,025.00	1000	ea	$3.03
OpenTrons 200µL filter tips	Library Prep	1	$687.50	100	ea	$6.88
OpenTrons 20 µL tips	Library Prep	1	$3,025.00	1000	ea	$3.03
OpenTrons 20 µL filter tips	Library Prep	2	$687.50	100	ea	$13.75
					Subtotal:	$369.77
NextSeq 550	Sequencing	11	$5270.00	110	Gbp	$527.00
					Subtotal:	$527.00
**Per-sample costs**						
Extraction	$0.64					
Library Prep	$3.85					
Sequencing	$5.49					
Total	$9.98					
Per HQ genome, no cull	$26.23					
Per HQ genome, cull after extraction	$18.21					
Per HQ genome, cull after library	$16.55					
