# Supplementary material for: A low-cost genomics workflow enables isolate screening and strain-level analyses within microbiomes
Source: Genome Biol. 2022 Oct 12;23:212. doi: 10.1186/s13059-022-02777-w (PMC9558970; doi:10.1186/s13059-022-02777-w)
Supplement: Supplementary file 4 — Additional file 4. Review History. [file 13059_2022_2777_MOESM4_ESM.docx]

Review History

**First round of review**

**Reviewer 1**

**Were you able to assess all statistics in the manuscript, including the appropriateness of statistical tests used?** Yes.

**Were you able to directly test the methods?** No.

**Comments to author:**

This paper describes a low cost open source genome-sequencing pipeline for bacterial isolates. The sequencing pipeline itself is likely to be of broad interest and utility, although it delivers high-quality genomes for a minority of isolates.

Comparisons with other comparable approaches should be included, e.g. https://pubmed.ncbi.nlm.nih.gov/34930397 which claims to achieve similar goals at similar costs.

The paper is compromised by the approaches to culture and bioinformatics analysis, which detract from the utility of the sequencing pipeline itself.

The approach to culture is clearly very restrictive in that they recover isolates largely or entirely restricted to a single phylum, the Firmicutes, which is not representative of this gut microbiome in general and in particular clearly misses out on many culturable Gram-negative bacteria from the phyla Proteobacteria and Bacteroidetes which are major components of this microbiome. Take a look at just a handful of the NCBI's automated analyses of chimp gut metagenomes, including one of their own, to see the obvious abundance of E. coli, Bacterioides, Prevotella etc in this habitat:
https://trace.ncbi.nlm.nih.gov/Traces/sra/?run=ERR2145011&krona=on
https://trace.ncbi.nlm.nih.gov/Traces/sra/?run=SRR12576917&krona=on
https://trace.ncbi.nlm.nih.gov/Traces/sra/?run=SRR12576918&krona=on

They should therefore remove any claims at having developed a high-throughput representative isolation approach and stick just to describing the genome sequencing workflow.

Similarly, they have used a highly unsophisticated approach to bioinformatics analysis, running an off-the-shelf pipeline without critically analyzing parameters or results. For example, it is unclear what version of GTDB is embedded within the just-turn-the-handle Bactopia pipeline. But if it is not R207, then it is outdated.

But more problematic still is what they say they have found. They report having cultured and genome-sequenced Anaplasma phagocytophilum, which is an obligate intracellular pathogen!
This casts doubt on all their findings.

In addition, the results presented in Figure 3 refer to various species clusters, but do not provide any kind of context as to what these clusters are (known species? new species?)

If this is a methods paper, then there is no need to include contentious and bitty downsream analyses of genomes.
The preferred option would thus be to restrict the outputs of their sequencing pipeline to sequencing stats, without further analyses.
But if they want to include phylogenomic analyses, these should be done to the highest standards using up-to-date tools and databases.

Note their genome sequences are not available as claimed here: https://qiita.ucsd.edu/study/description/14410
They are available at the EBI, but not at NCBI. However, as they fail to link genomes to EBI accession numbers it is impossible to corroborate or trouble shoot the bioinformatics analyses

**Reviewer 2**

**Were you able to assess all statistics in the manuscript, including the appropriateness of statistical tests used?** Yes.

**Were you able to directly test the methods?** No.

**Comments to author:**

The manuscript "Low-cost genomics enable high-throughput isolate screening and strain-level microbiome profiling" by Sanders et al. describes a low-cost, automated workflow for the isolation of bacterial cultures from fecal microbiome samples, which is showcased for 1911 isolates from 10 fecal samples from chimpanzees and bonobos, which are sequenced, assembled, classified (GTDB-Tk), and analyzed based on genome comparison (ANI), and plasmid populations that are identified in the assemblies. The manuscript estimates the cost to generate these isolate genome assemblies from fecal samples.

The protocol, its description and validation, are interesting and relevant for microbiome researcher and comparative genomics, and the manuscript seems suitable for the journal Genome Biology. However, I am concerned about the broader applicability of the presented protocol, and I think the presented isolate genome sequence analysis should be expanded to convince readers of the importance of the protocol. Finally, the authors should more clearly state the potential and limitations of their protocol and findings.

Major comments:
1. How easily would the presented protocol be applicable to other labs? It heavily relies on the OpenTrons OT-2 robot system and the custom-made 3D-printed lab equipment, and one of the authors is the founder of a lab equipment company.
- How adaptable is the protocol to other, alternative lab environments, including other robotic systems or lab equipment?
- What are the expected (monetary and developmental) requirements for other labs to implement a similar setup?

2. The manuscript makes strong statements about the utility of their protocol for microbiome analysis, but it is based on a limited and not fully convincing biological analysis.
- In the Results, the authors should more clearly demonstrate novel findings in the "Isolate population genetics" chapter, which is not very clear and convincing and could be expanded. As far as I understand, it only describes clusters of related genomes with different degrees of genomic heterogeneity, but this is not analyzed or presented in the context of inter and intra-sample or -species diversity, although these diversity metrics would be very interesting to better understand microbiota organization and function. And the presented findings are needed to justify the new proposed protocol.
- The presentation of the "genome-wide recombination rates" is unclear: what are those plots exactly showing and why is that relevant for a species, a microbiome, or the utility of the protocol?

3. Based on the previous point, the description and discussion of the protocol and findings should be adapted.
- All recovered isolates are from the phylum Firmicutes, so not really representative of the entire microbiota. The protocol therefore would have to be adapted for general microbiome analysis or seems more useful for targeted comparative genome analyses of microbiota fractions - which is great but should be described as such.
- The presented data do not justify the statements that the new method "circumvents the need for isolate screening" (Abstract) or "alleviates the need for 16S rDNA- or mass spectrometry-based approaches". With the presented data, it seems only useful for specific bacterial taxa and project interests.
- The manuscript presents the cultivation from samples that were stored in RNAlater (which I thought kills all bacteria), and for more than 10 years, which is very impressive and interesting, and might explain some of the taxonomic bias of the isolation. I would recommend to discuss this aspect of the manuscript more thoroughly and prominently.


Other comments:
- Abstract: It's not just species from "in the chimpanzee gut microbiota".
- Why was the Qiagen PowerSoil DNA extraction kit used, which seems unusual? Can you provide a justification or reference?
- Page 7, "Sequence analysis": Rephrase and split first sentence for clarity.
- Page 8, Methods: Add a short explanation of how genomes and plasmids are linked.
- Page 8: "Of the fully-assembled isolate genomes,..." - how many are those?
- Page 9: "Claustridium"
- Page 9: "Sourmash was able to classify more of the samples" - what means "samples" here?
- Page 10: "375 isolate assemblies yielded..." - What does that mean here, 375 samples or clones? Of how many in total?
- Discussion, Page 11: "costs of $10 per bacterium" contradicts the $20 described in the Results
- Page 11: "which IN yielded"
- Figure 2: Explain numbers and highlighted clusters in the center of the figure.
- Figure 3 needs more information, such as a legend for the heatmaps and a more detailed description of the recombination plots ("Recombination starts")

**Reviewer 3**

**Were you able to assess all statistics in the manuscript, including the appropriateness of statistical tests used?** No.

**Were you able to directly test the methods?** Yes.

**Comments to author:**

It was nice to read all details about the new workflow and the authors present a showcase (exchange of genetic information) that will also be of interest. Most of all, I want to thank the authors for such a transparency about their method and making all materials and data available. This is exemplary.

1/ The main recommendation I have is to decrease the selling factor within this paper. The title sounds very attractive; it gathers all the buzz words one may want to have: low-cost, high-throughput, isolates, microbiome, strain-level. However, the details given throughout the text… (again, it was really appreciated that the authors made efforts to provide a very clear and comprehensive manuscript)… reveal a substantially different story. The reality would necessitate the following amendment at the end of the title, which I understand is less attractive: "… for a limited number of mostly aerobic bacterial species".
The authors used samples that were stored for a long period of time; using storage conditions that selected certain bacteria; the real diversity out of 1,911 isolates is eventually 45 species, representing a cultured fraction of 3% of the original ecosystem (if I understood the values presented in the paper correctly). Despite the use of an anaerobic chamber, the majority of taxa obtained are not strictly anaerobic species (this will have to be clearly stated in the revised version). I feel all these elements must be made much clearer to the readers, already in the title and abstract. To me, the following title sounds more appropriate and a good fit to this journal "A low-cost genomic workflow enables population genetic analyses within microbiomes". I took the title as an example, but please pay attention to this (inflated selling factor) throughout the manuscript. Another example, in the discussion, is the use of the term "high-throughput bacterial isolation", which is misleading as it clearly contradicts the low depth of isolation mentioned above. There are other examples like this in the manuscript.

2/ Besides the mapping of metagenomic reads to the genome assemblies (average 2.97%), I think it is important to estimate the fraction of taxa present in the original ecosystem that were covered by isolates sequences captured by the method (e.g., at different taxonomic ranks, e.g. phyla, families, genera, species).

3/ In somewhat a similar line of thoughts, regarding the plasmids, it would be nice to have an estimate of the yet-unknown fraction of them obtained in this study comparing to existing databases.
I also wonder whether, again, the low diversity of species obtained should not be mentioned as a clear potential confounder of the analysis about the distribution of plasmids.
In this section (page 10), I think it will also be important to be precise with respect to the term "taxa"; which taxonomic rank is meant exactly?

4/ Points pertaining to the workflow relevance, throughput, cost, and transferability:
a/ A total of 1,911 were collected and analysed by two persons with "just a few months"; please indicate a more precise period of time. Moreover, it will be important to know the depth of isolation (number of isolates and species) per sample (mean +/- sd)
b/ By reading the experimental details, it became clear that, after original plating, colonies are directly transferred for further processing. Classical approaches include re-streaking to ensure purity, which I understand is not feasible considering the aim of the study to be high-throughput. The price to pay is apparently to accept a fraction of 10% mixed cultures.
Altogether, I actually wonder what type of controls/blanks the authors used during the multiple steps of their workflow to control for potential contaminations?
Moreover, looking at the data in figure 1, the loss due to mixed cultures mentioned above is complemented by other sources of loss, which altogether account for up to approx. half of the isolates (ca. 600) and their data that cannot be used. Even at an optimal price per isolate (see next comment), this is a substantial price to pay. For the sake of transparency, I think it will be important to emphasize this in the manuscript.
c/ The low-cost put in the forefront by the authors (ca. USD10) will not hold true in most places I know; sequencing costs will be much higher than USD3 per genomes.
d/ Again, releasing all details about the workflow, including information on specific 3D-printed components, was highly appreciated. But exactly this part (need of custom-made material) makes it a little more complicated to transfer the workflow.
e/ I understand that generating both the genome and 16S rRNA gene sequences of isolates represents redundancy for known taxa, hence justifying the choice of the authors to go only for genomes. However, for isolates representing novel species, which I suppose the authors will end up gathering at some point with their workflow, this is problematic. It will be detrimental on the long term to release data about potentially new bacteria without having them linked to 16S rRNA gene sequences. Failure to generate high-quality 16S rRNA gene sequences from genome sequences is not a rare event. This may be commented by the authors in the paper and potentially presented as an additional limitation.

5/ Additional points:
a/ It seems that only raw data were submitted for the genomes. If correct, the assemblies generated by the authors must also be deposited.
b/ Consistently use the term "16S rRNA gene" (not 16S rDNA) and RELATIVE abundance.
c/ page 9, Clostridium instead of Claustridium?

**Authors Response**

Dear editor,

We would like to thank you and the Reviewers for your time and constructive comments on our manuscript. In this revision, we have addressed all comments from the Reviewers, leading to numerous edits throughout the manuscript and several new analyses. These changes have significantly improved the manuscript. We have rewritten the introduction, results, and discussion to better highlight the strengths and limitations of our approach. We have also conducted several new population genetic analyses that better demonstrate that isolate genomes provide utility for microbiota research beyond that provided by metagenomic sequencing alone. In particular, new analyses of plasmid distributions among individual bacterial and primate hosts revealed significant divergence of homologous plasmids between chimpanzee and bonobo subspecies. Below we list all of the Reviewers’ comments followed by our responses in **bold**.

Reviewer reports:

Reviewer #1: This paper describes a low cost open source genome-sequencing pipeline for bacterial isolates. The sequencing pipeline itself is likely to be of broad interest and utility, although it delivers high-quality genomes for a minority of isolates.

**We thank the reviewer for these comments.**

Comparisons with other comparable approaches should be included, e.g. <https://pubmed.ncbi.nlm.nih.gov/34930397> which claims to achieve similar goals at similar

**We have added to the text a more thorough comparison between our method and existing approaches. One of the primary motivations for the development of our protocol was to reduce as much as possible the capital costs needed to sequence isolate genomes in a high-throughput manner. The cited article uses a range of specialized equipment, including a high-end liquid handling robot, whereas our workflow uses equipment that can be purchased and 3-D-printed for <$10,000. We have added the suggested citation, and added language to the introduction and conclusion to emphasize the points about capital costs.**

The paper is compromised by the approaches to culture and bioinformatics analysis, which detract from the utility of the sequencing pipeline itself.

**We have made a number of changes to address these problems; please see below.**

The approach to culture is clearly very restrictive in that they recover isolates largely or entirely restricted to a single phylum, the Firmicutes, which is not representative of this gut microbiome in general and in particular clearly misses out on many culturable Gram-negative bacteria from the phyla Proteobacteria and Bacteroidetes which are major components of this microbiome. Take a look at just a handful of the NCBI's automated analyses of chimp gut metagenomes, including one of their own, to see the obvious abundance of E. coli, Bacterioides, Prevotella etc in this habitat:

<https://trace.ncbi.nlm.nih.gov/Traces/sra/?run=ERR2145011&krona=on>

<https://trace.ncbi.nlm.nih.gov/Traces/sra/?run=SRR12576917&krona=on>

<https://trace.ncbi.nlm.nih.gov/Traces/sra/?run=SRR12576918&krona=on>

They should therefore remove any claims at having developed a high-throughput representative isolation approach and stick just to describing the genome sequencing workflow.

**We agree with the reviewer that this manuscript presents a genome sequencing workflow rather than a cultivation and sequencing workflow. We have made a number of revisions to clarify this point. This includes changing the title and removing references to the cultivation steps in portions of the text describing the specific contributions of the paper.**

**Additionally, we have added text in the methods and discussion (second to last paragraph) emphasizing that the storage conditions of the samples mean that the remaining population of viable cells should not be taken as representative of the original population.**

Similarly, they have used a highly unsophisticated approach to bioinformatics analysis, running an off-the-shelf pipeline without critically analyzing parameters or results. For example, it is unclear what version of GTDB is embedded within the just-turn-the-handle Bactopia pipeline. But if it is not R207, then it is outdated.

**A pipeline like Bactopia is a typical way to generate useful data about large numbers of genomes, and likely representative of a typical 'first pass' analysis of many users. We believe it is appropriate for demonstration purposes, illustrating the genomic output of the sequencing pipeline, as this is primarily a manuscript about the method that we developed.**

**We have updated the text to indicate the version of GTDB used by the pipeline, which was the most current available when the analyses were performed (R207 was released just two weeks prior to the submission of the manuscript). Note that none of the results presented here depend on differences between versions of GTDB.**

But more problematic still is what they say they have found. They report having cultured and genome-sequenced Anaplasma phagocytophilum, which is an obligate intracellular pathogen!

This casts doubt on all their findings.

**We have added text to the results highlighting the fact that the kmer MinHash approach used by SourMash, which generated the single Anaplasma annotation, is likely to be less accurate than the more stringent GTDB marker gene approach. We now emphasize that MinHash annotations should be interpreted with caution, and favor the annotations provided by GTDB.**

In addition, the results presented in Figure 3 refer to various species clusters, but do not provide any kind of context as to what these clusters are (known species? new species?)

**We have clarified the clustering methodology in the main text and updated the figure caption to provide additional clarity. In addition, we have moved the figures showing intraspecific genomic variation to Figure 2 in order to provide phylogenetic context for these examples.**

If this is a methods paper, then there is no need to include contentious and bitty downsream analyses of genomes.

The preferred option would thus be to restrict the outputs of their sequencing pipeline to sequencing stats, without further analyses.

But if they want to include phylogenomic analyses, these should be done to the highest standards using up-to-date tools and databases.

**As this is a methods paper, we agree that extensive analysis of the genomes generated is unnecessary. However, as noted by other reviewers, some analysis is useful for descriptive purposes and to highlight the utility of genome sequencing as an alternative or complement to marker-gene or metagenomic sequencing. Thus we have trimmed some of the genomic analysis (specifically removing the confusing recombination analysis and figure) while strengthening others. Importantly, we have replaced the ad-hoc estimate of plasmid diversity presented in the original manuscript with a more rigorous analysis using the state-of-the-art PlasX prediction pipeline.**

Note their genome sequences are not available as claimed here: <https://qiita.ucsd.edu/study/description/14410>

They are available at the EBI, but not at NCBI. However, as they fail to link genomes to EBI accession numbers it is impossible to corroborate or trouble shoot the bioinformatics analyses

**We have added the completed genome assemblies to the Qiita study, as well as updated Table S2 with per-isolate EBI accession numbers.**

Reviewer #2: The manuscript "Low-cost genomics enable high-throughput isolate screening and strain-level microbiome profiling" by Sanders et al. describes a low-cost, automated workflow for the isolation of bacterial cultures from fecal microbiome samples, which is showcased for 1911 isolates from 10 fecal samples from chimpanzees and bonobos, which are sequenced, assembled, classified (GTDB-Tk), and analyzed based on genome comparison (ANI), and plasmid populations that are identified in the assemblies. The manuscript estimates the cost to generate these isolate genome assemblies from fecal samples.

**We thank the reviewer for their time and helpful comments.**

The protocol, its description and validation, are interesting and relevant for microbiome researcher and comparative genomics, and the manuscript seems suitable for the journal Genome Biology. However, I am concerned about the broader applicability of the presented protocol, and I think the presented isolate genome sequence analysis should be expanded to convince readers of the importance of the protocol. Finally, the authors should more clearly state the potential and limitations of their protocol and findings.

**We agree with these concerns raised by the reviewer. We have revised the title, abstract, and main text of the paper to emphasize that the novelty of the method is in the genome sequencing protocol rather than cultivation protocols. We have also added text clarifying how our protocol compares to other previously-published protocols for high-throughput sequencing.**

**Additionally, we have removed uninformative analyses (e.g., recombination scan, formerly Figure 3 and Figure S5), added an analysis of 16S rRNA gene sequences recovered from assemblies, and updated the analysis of plasmid diversity using a more thorough, published protocol.**

Major comments:

1. How easily would the presented protocol be applicable to other labs? It heavily relies on the OpenTrons OT-2 robot system and the custom-made 3D-printed lab equipment, and one of the authors is the founder of a lab equipment company.

- How adaptable is the protocol to other, alternative lab environments, including other robotic systems or lab equipment?

- What are the expected (monetary and developmental) requirements for other labs to implement a similar setup?

**Our goal with this protocol was specifically to lower the barriers to adoption by other labs, which guided many of our decisions pertaining to equipment. The only equipment that would be difficult to substitute would be the OpenTrons liquid handling robot, which is already the most inexpensive liquid handling robot on the market. Although the protocol could certainly be adopted to other instruments, a substantial amount of investment would need to be made to do so.**

**For the home-made equipment we used, we have added a table of commercially available alternatives.**

**Our best estimates in terms of total necessary capital outlay are included in the "Protocol cost estimates" subheading under Results.**

2. The manuscript makes strong statements about the utility of their protocol for microbiome analysis, but it is based on a limited and not fully convincing biological analysis.

- In the Results, the authors should more clearly demonstrate novel findings in the "Isolate population genetics" chapter, which is not very clear and convincing and could be expanded. As far as I understand, it only describes clusters of related genomes with different degrees of genomic heterogeneity, but this is not analyzed or presented in the context of inter and intra-sample or -species diversity, although these diversity metrics would be very interesting to better understand microbiota organization and function. And the presented findings are needed to justify the new proposed protocol

**We thank the reviewer for this excellent suggestion to further examine our data in the context of our sample diversity. We have added new analyses of plasmid distributions that show significant divergence of homologous plasmid sequences between chimpanzee and bonobo host species (Fig. 4). These results greatly improve the manuscript by providing a clear example of an analysis and biological insight made possible by isolate genomics.**

- The presentation of the "genome-wide recombination rates" is unclear: what are those plots exactly showing and why is that relevant for a species, a microbiome, or the utility of the protocol?

**We agree that these were superfluous, and have removed them from the manuscript.**

3. Based on the previous point, the description and discussion of the protocol and findings should be adapted.

- All recovered isolates are from the phylum Firmicutes, so not really representative of the entire microbiota. The protocol therefore would have to be adapted for general microbiome analysis or seems more useful for targeted comparative genome analyses of microbiota fractions - which is great but should be described as such.

**We have changed the title, abstract, and introduction to clarify that the focus of this manuscript is as a genome sequencing tool rather than a cultivation pipeline. The taxonomic limitations in this specific sample set are not a product of the sequencing protocol, but of the samples themselves. We have added language clarifying this where taxonomy results are presented.**

**Any cultivation-based approach necessarily involves biased sampling. We have edited the text to clarify that our method is not an alternative to less-biased methods such as shotgun metagenomic sequencing, but rather a more data-rich approach for culture-based investigations. We have added a sentence in the introduction ("Although cultivation necessarily introduces bias in the specific taxa that are recovered, and thus cannot replace metagenomic methods for understanding microbial communities, it offers a few clear advantages.") and changed the title to clarify these points.**

- The presented data do not justify the statements that the new method "circumvents the need for isolate screening" (Abstract) or "alleviates the need for 16S rDNA- or mass spectrometry-based approaches". With the presented data, it seems only useful for specific bacterial taxa and project interests.

**We have edited the title and text of the manuscript to help to clarify these points. Our method does not represent a potential substitute for community analyses using untargeted rRNA gene or metagenomic survey methods. However, it does alleviate the need for screening isolates using 16S rDNA sequencing before whole-genome sequencing, as is commonly performed in order to reduce costs of sequencing redundant genomes. Here, the low cost at which we can generate whole genomes from bacterial isolates alleviates the need for dereplication based on 16S rDNA screening.**

- The manuscript presents the cultivation from samples that were stored in RNAlater (which I thought kills all bacteria), and for more than 10 years, which is very impressive and interesting, and might explain some of the taxonomic bias of the isolation. I would recommend to discuss this aspect of the manuscript more thoroughly and prominently.

**We agree that the suboptimal sample preservation is likely the root cause of the limited taxonomic breadth we were able to recover, and have added language throughout the manuscript emphasizing this point.**

Other comments:

- Abstract: It's not just species from "in the chimpanzee gut microbiota".

**We have updated the manuscript throughout to reflect that samples came from both chimpanzees and bonobos.**

- Why was the Qiagen PowerSoil DNA extraction kit used, which seems unusual? Can you provide a justification or reference?

**The Qiagen (formerly MoBio) PowerSoil kit is among the most commonly-used DNA extraction kits in microbiome research; it is the kit used in the standard Earth Microbiome Project extraction protocols.** [**http://earthmicrobiome.org/protocols-and-standards/dna-extraction-protocol/**](http://earthmicrobiome.org/protocols-and-standards/dna-extraction-protocol/)

- Page 7, "Sequence analysis": Rephrase and split first sentence for clarity.

**We have edited this sentence.**

- Page 8, Methods: Add a short explanation of how genomes and plasmids are linked.

**We linked plasmids and genomes through the isolation of individual bacterial lineages in pure culture. We have edited the text to better clarify this advantage of isolate genomics over metagenomic data alone.**

- Page 8: "Of the fully-assembled isolate genomes,..." - how many are those?

**We have added this information to this sentence.**

- Page 9: "Claustridium"

**We have edited this sentence.**

- Page 9: "Sourmash was able to classify more of the samples" - what means "samples" here?

**We have edited this sentence to read “isolates” instead of “samples”.**

- Page 10: "375 isolate assemblies yielded..." - What does that mean here, 375 samples or clones? Of how many in total?

**We have updated this sentence to clarify that all isolate assemblies were scanned using PlasX, but plasmids were identified from only a subset of them.**

- Discussion, Page 11: "costs of $10 per bacterium" contradicts the $20 described in the Results

**We have edited this sentence to clarify that $10 refers to the marginal cost.**

- Page 11: "which IN yielded"

**We have edited this sentence.**

- Figure 2: Explain numbers and highlighted clusters in the center of the figure.

**We have combined the example heatmaps with this figure (now panel B) in order to more clearly link the three highlighted clades with diversity heatmaps. We have also updated the legend to clarify.**

- Figure 3 needs more information, such as a legend for the heatmaps and a more detailed description of the recombination plots ("Recombination starts")

**The heatmaps from Figure 3 have been moved to Figure 2, and we have omitted the confusing recombination analysis from the manuscript.**

Reviewer #3: It was nice to read all details about the new workflow and the authors present a showcase (exchange of genetic information) that will also be of interest. Most of all, I want to thank the authors for such a transparency about their method and making all materials and data available. This is exemplary.

**We very much appreciate the reviewer's time and helpful suggestions.**

1/ The main recommendation I have is to decrease the selling factor within this paper. The title sounds very attractive; it gathers all the buzz words one may want to have: low-cost, high-throughput, isolates, microbiome, strain-level. However, the details given throughout the text… (again, it was really appreciated that the authors made efforts to provide a very clear and comprehensive manuscript)… reveal a substantially different story. The reality would necessitate the following amendment at the end of the title, which I understand is less attractive: "… for a limited number of mostly aerobic bacterial species".

The authors used samples that were stored for a long period of time; using storage conditions that selected certain bacteria; the real diversity out of 1,911 isolates is eventually 45 species, representing a cultured fraction of 3% of the original ecosystem (if I understood the values presented in the paper correctly). Despite the use of an anaerobic chamber, the majority of taxa obtained are not strictly anaerobic species (this will have to be clearly stated in the revised version). I feel all these elements must be made much clearer to the readers, already in the title and abstract. To me, the following title sounds more appropriate and a good fit to this journal "A low-cost genomic workflow enables population genetic analyses within microbiomes". I took the title as an example, but please pay attention to this (inflated selling factor) throughout the manuscript. Another example, in the discussion, is the use of the term "high-throughput bacterial isolation", which is misleading as it clearly contradicts the low depth of isolation mentioned above. There are other examples like this in the manuscript.

**We agree that we missed with the description of the study’s contributions in the initially-submitted title and abstract, and have made changes accordingly. We have edited the title, abstract, and manuscript to more accurately reflect the primary contribution of the study, which is (see also responses to reviewers 1 and 2) primarily a genome sequencing pipeline for isolates, rather than a cultivation method.**

**We have also added text clarifying and emphasizing the samples employed, and that their preservation in RNALater is likely behind the limited taxonomic composition of the cultured isolates.**

2/ Besides the mapping of metagenomic reads to the genome assemblies (average 2.97%), I think it is important to estimate the fraction of taxa present in the original ecosystem that were covered by isolates sequences captured by the method (e.g., at different taxonomic ranks, e.g. phyla, families, genera, species).

**We have rewritten the manuscript to better emphasize that cultivation was not expected to reflect the full taxonomic breadth of the original samples due to loss of viability in the RNALater preservative. Given the revised focus of this resubmission, we decided against reporting extensive details on the fraction of microbial taxa in the sample represented in cultured isolates.**

3/ In somewhat a similar line of thoughts, regarding the plasmids, it would be nice to have an estimate of the yet-unknown fraction of them obtained in this study comparing to existing databases.

**This is a great idea, and we have completely reworked the plasmid analysis to use a state-of-the-art plasmid prediction and analysis pipeline, PlasX (see** [**https://www.biorxiv.org/content/10.1101/2020.11.01.361691v2.full**](https://www.biorxiv.org/content/10.1101/2020.11.01.361691v2.full)**). In addition, as suggested, we have compared all predicated plasmids against the PLSDB plasmid sequence database to estimate overall novelty of the recovered plasmid sequences.**

I also wonder whether, again, the low diversity of species obtained should not be mentioned as a clear potential confounder of the analysis about the distribution of plasmids.

**Text edit. We agree, and in restructuring the plasmid analysis have deemphasized inferences about plasmid distribution. We have also mentioned the issue of limited sample size for such analyses in the discussion, third paragraph.**

In this section (page 10), I think it will also be important to be precise with respect to the term "taxa"; which taxonomic rank is meant exactly?

**We have updated the text to specify family-level rank.**

4/ Points pertaining to the workflow relevance, throughput, cost, and transferability:

a/ A total of 1,911 were collected and analysed by two persons with "just a few months"; please indicate a more precise period of time. Moreover, it will be important to know the depth of isolation (number of isolates and species) per sample (mean +/- sd)

**We have removed the reference to time effort spent because we felt it was difficult to provide meaningful context, as this project was one of many being concurrently performed in the lab, and it was also done largely during the unique situations associated with the early pandemic.**

**We have added means and standard deviations for isolates and species (i.e. unique GTDB taxon strings) recovered per host individual in the text.**

b/ By reading the experimental details, it became clear that, after original plating, colonies are directly transferred for further processing. Classical approaches include re-streaking to ensure purity, which I understand is not feasible considering the aim of the study to be high-throughput. The price to pay is apparently to accept a fraction of 10% mixed cultures.

**We have added a note to the discussion section, in the paragraph where we discuss possible improvements to the protocol, indicating that re-streaking could decrease contamination.**

Altogether, I actually wonder what type of controls/blanks the authors used during the multiple steps of their workflow to control for potential contaminations?

**We have added a sentence to the first paragraph of the Cultivation section in Methods describing our use of blank control plates during anaerobic cultivation.**

Moreover, looking at the data in figure 1, the loss due to mixed cultures mentioned above is complemented by other sources of loss, which altogether account for up to approx. half of the isolates (ca. 600) and their data that cannot be used. Even at an optimal price per isolate (see next comment), this is a substantial price to pay. For the sake of transparency, I think it will be important to emphasize this in the manuscript.

**We have clarified this point in the text. We have also added lines in the supplemental cost calculation table that show the per-high-quality-assembly cost for the protocol as executed, as well as accounting for various levels of winnowing at different steps in the protocol.**

c/ The low-cost put in the forefront by the authors (ca. USD10) will not hold true in most places I know; sequencing costs will be much higher than USD3 per genomes.

**The sequencing costs we have indicated in Table S3 (about $48/Gbp) are not unusual for academic laboratories. For example, these are very similar to prices charged to academic users by the UC Davis Genome Center for the same NextSeq flow cell that we used. Even when factoring in their external rates, the total cost per genome only rises to about $13. Furthermore, choosing a lower cost/base sequencing platform like a NovaSeq S1 has the potential to lower costs even more ($36/Gbp for UC Davis external academic users, $25/Gbp for UC internal users).**

d/ Again, releasing all details about the workflow, including information on specific 3D-printed components, was highly appreciated. But exactly this part (need of custom-made material) makes it a little more complicated to transfer the workflow.

**We designed our 3D-printed components specifically to decrease the required capital costs for labs to adopt these sorts of protocols; however, commercial alternatives are available for each of these components. We have added a supplemental table including links to example devices for each. This addition substantially reduces complication of transferring the workflow to a new lab.**

e/ I understand that generating both the genome and 16S rRNA gene sequences of isolates represents redundancy for known taxa, hence justifying the choice of the authors to go only for genomes. However, for isolates representing novel species, which I suppose the authors will end up gathering at some point with their workflow, this is problematic. It will be detrimental on the long term to release data about potentially new bacteria without having them linked to 16S rRNA gene sequences. Failure to generate high-quality 16S rRNA gene sequences from genome sequences is not a rare event. This may be commented by the authors in the paper and potentially presented as an additional limitation.

**We have added sections (Methods/Sequence Analysis and Results/Isolate population genetics) detailing the recovery of 16S rRNA genes from the assembled isolate genomes using a dedicated, peer-reviewed tool (PhyloFlash, gruber-Vodica, Seah & Pruesse). We have also included some specific information detailing the observed estimated Average Nucleotide Identity between genomes sharing identical 16S rRNA gene haplotypes.**

5/ Additional points:

a/ It seems that only raw data were submitted for the genomes. If correct, the assemblies generated by the authors must also be deposited.

**We have added the genome assemblies to the Qiita repository as well.**

b/ Consistently use the term "16S rRNA gene" (not 16S rDNA) and RELATIVE abundance.

**All references to “16S” and “abundance” have been changed to “16S rRNA gene” and “relative abundance”**

c/ page 9, Clostridium instead of Claustridium?

**Thank you. We have corrected this error.**

**Second round of review**

**Reviewer 2**

The authors have addressed my comments as well as those of the other reviewers.

My only remaining concern is about Fig. 3 and its legend. The legend seems not to match the figure:
- I don't see any "diamond-shaped nodes" (contigs), only circles (isolates)
- colors seem to refer to the pan hosts, not the isolate taxonomic background
- the color bars on the right of B-E are not explained (.991-1.00)
- etc.

That needs to be fixed!

**Reviewer 2**

Thanks for the changes made to the manuscript. I think it can be accepted for publication. I have only a few minor comments to add:

- Page 1, L39, first sentence of the introduction. I argue this is the definition of microbiota/microbiome, and not a metagenome, which is the assemblage of individual genomes within an ecosystem. The sentence of the authors clearly refers to the organisms, not their genomes.
- “For this reason, cultivation has seen renewed interest, even earning its own ‘omics’ appellation: high throughput approaches to culturing, or ‘culturomics’ [10].” I think that the term ‘culturomics’ is not helpful; even detrimental to the field. It was coined by a group, which generated a lot of chaos in bacterial taxonomy and nomenclature throughout years of aggressive lobbying. The throughput of the ‘culturomics’ approach is solely based on parallelized manual work in dozens of anaerobic boxes, which does not represent a major technical advance compared with work from the 1960’s. The use of MALDI-biotyping was instrumental in the ‘culturomics’ approach, which might be the only merit of their workflow. I would encourage the authors to not spread the term ‘culturomics’; their own workflow is much more innovative than ‘culturomics’ will ever be.
- “Individual colonies were picked from plates into 900 μL of liquid media (Table S3) using a sterile wooden toothpick.” I suppose this was done manually? Please clarify in the sentence.
- I still think that, the fact the authors used an anaerobic workstation to eventually recover mostly aerobes, microaerophiles, or facultative/aerotolerant anaerobes does not come across clearly enough in the manuscript. It is actually not clear which steps were performed under anaerobic conditions (e.g., methods; Fig 1 does not picture/state this either). Considering that (i) previous studies reported a substantial fraction of human gut microbiota being represented by spore formers (assuming it is sound to extrapolate this to wild-living chimpanzees and bonobos) (PMID: 27144353), and (ii) such bacteria are likely to survive the storage conditions used by the authors, I would have expected a higher number of strict anaerobes being recovered. Hence, the question/concern whether the workflow presented is indeed appropriate for strict anaerobes. I think amending the methods in this respect and adding a corresponding note to the discussion when mentioning limitations with help clarifying this for the readers.

**Authors Response**

**Point-by-point responses to the reviewers’ comments:**

Reviewer #3: Thanks for the changes made to the manuscript. I think it can be accepted for publication. I have only a few minor comments to add:

- Page 1, L39, first sentence of the introduction. I argue this is the definition of microbiota/microbiome, and not a metagenome, which is the assemblage of individual genomes within an ecosystem. The sentence of the authors clearly refers to the organisms, not their genomes.

**We agree and have made the change.**

- "For this reason, cultivation has seen renewed interest, even earning its own 'omics' appellation: high throughput approaches to culturing, or 'culturomics' [10]." I think that the term 'culturomics' is not helpful; even detrimental to the field. It was coined by a group, which generated a lot of chaos in bacterial taxonomy and nomenclature throughout years of aggressive lobbying. The throughput of the 'culturomics' approach is solely based on parallelized manual work in dozens of anaerobic boxes, which does not represent a major technical advance compared with work from the 1960's. The use of MALDI-biotyping was instrumental in the 'culturomics' approach, which might be the only merit of their workflow. I would encourage the authors to not spread the term 'culturomics'; their own workflow is much more innovative than 'culturomics' will ever be.

**We agree fully, and appreciate the vote of confidence! We have removed the reference to 'culturomics', and instead refer more generally to 'automation and screening techniques'.**

- "Individual colonies were picked from plates into 900 μL of liquid media (Table S3) using a sterile wooden toothpick." I suppose this was done manually? Please clarify in the sentence.

**We have edited this sentence for clarity.**

- I still think that, the fact the authors used an anaerobic workstation to eventually recover mostly aerobes, microaerophiles, or facultative/aerotolerant anaerobes does not come across clearly enough in the manuscript. It is actually not clear which steps were performed under anaerobic conditions (e.g., methods; Fig 1 does not picture/state this either). Considering that (i) previous studies reported a substantial fraction of human gut microbiota being represented by spore formers (assuming it is sound to extrapolate this to wild-living chimpanzees and bonobos) (PMID: 27144353), and (ii) such bacteria are likely to survive the storage conditions used by the authors, I would have expected a higher number of strict anaerobes being recovered. Hence, the question/concern whether the workflow presented is indeed appropriate for strict anaerobes. I think amending the methods in this respect and adding a corresponding note to the discussion when mentioning limitations with help
clarifying this for the readers.

**All cultivaiton steps, from inoculation to regrowth in liquid media, took place under anaerobic conditions. We have clarified this in the methods, and added a sentence to the discussion making note specifically of the lack of strict anaerobes and our inability to discern what specifically accounts for their absence.**

Reviewer #2: The authors have addressed my comments as well as those of the other reviewers.

My only remaining concern is about Fig. 3 and its legend. The legend seems not to match the figure:
- I don't see any "diamond-shaped nodes" (contigs), only circles (isolates)
- colors seem to refer to the pan hosts, not the isolate taxonomic background
- the color bars on the right of B-E are not explained (.991-1.00)
- etc.

That needs to be fixed!

**We apologize for the oversight. We have updated the figure caption to correspond accurately to the new figure.**
